# Characterization of Danaparoid Complex Extractive Drug by an Orthogonal Analytical Approach

**DOI:** 10.3390/molecules22071116

**Published:** 2017-07-05

**Authors:** Cristina Gardini, Elena Urso, Marco Guerrini, René van Herpen, Pauline de Wit, Annamaria Naggi

**Affiliations:** 1 Centro Alta Tecnologia Istituto di Ricerche Chimiche e Biochimiche G. Ronzoni S.r.l., via G. Colombo 81, 20133 Milan, Italy; gardini@cat-ronzoni.it; 2 Istituto di Ricerche Chimiche e Biochimiche G. Ronzoni S.r.l., via G. Colombo 81, 20133 Milan, Italy; urso@ronzoni.it (E.U.); guerrini@ronzoni.it (M.G.); 3 Aspen Oss B.V., Kloosterstraat 6, 5349 AB Oss, The Netherlands; rvanherpen@nl.aspenpharma.com (R.v.H.); pdewit@nl.aspenpharma.com (P.d.W.)

**Keywords:** danaparoid sodium, low molecular weight glycosaminoglycans, orthogonal multi-analytical methods, sequence and compositional investigations, component quantitative analysis

## Abstract

Danaparoid sodium salt, is the active component of ORGARAN, an anticoagulant and antithrombotic drug constituted of three glycosaminoglycans (GAGs) obtained from porcine intestinal mucosa extracts. Heparan sulfate is the major component, dermatan sulfate and chondroitin sulfate being the minor ones. Currently dermatan sulfate and chondroitin sulfate are quantified by UV detection of their unsaturated disaccharides obtained by enzymatic depolymerization. Due to the complexity of danaparoid biopolymers and the presence of shared components, an orthogonal approach has been applied using more advanced tools and methods. To integrate the analytical profile, 2D heteronuclear single quantum coherence (HSQC) NMR spectroscopy was applied and found effective to identify and quantify GAG component signals as well as those of some process signatures of danaparoid active pharmaceutical ingredient (API) batches. Analyses of components of both API samples and size separated fractions proceeded through the determination and distribution of the molecular weight (Mw) by high performance size exclusion chromatographic triple detector array (HP-SEC-TDA), chain mapping by LC/MS, and mono- (^1^H and ^13^C) and bi-dimensional (HSQC) NMR spectroscopy. Finally, large scale chromatographic isolation and depolymerization of each GAG followed by LC/MS and 2D-NMR analysis, allowed the sequences to be defined and components to be evaluated of each GAG including oxidized residues of hexosamines and uronic acids at the reducing ends.

## 1. Introduction

Danaparoid sodium, constituted by a mixture of Low Molecular Weight (LMW) heparan sulfate (HS), dermatan sulfate (DS), and chondroitin sulfate (CS), extracted from porcine intestinal mucosa, is the active component of ORGARAN an anticoagulant and antithrombotic drug approved for prophylaxis of post-operative deep-vein thrombosis. Its beneficial effect upon factor IIa (thrombin) is shown by the anti-factor Xa/IIa ratio more than that of heparin [1,2,3].

A weight/weight (*w*/*w*) percentage maximum of 8.5% of CS and in the range of 8.0% up to 16.0% for DS, have been specified and quantified by an enzymatic method reported in the danaparoid monograph of the EC-Pharmacopoeia [4]. The chondroitinase selective depolymerization of galactosaminoglycans to UV detectable unsaturated disaccharides was originally developed to quantify CS and DS present in API heparin batches. Alternative more feasible NMR quantifications have been developed for the components of danaparoid API batches [5] and for heparin composition [6].

The linear polymers HS, CS, and DS are the most abundant mucopolysaccharides in the body and ubiquitous components of connective tissue and cartilages. As other extracted and purified glycosaminoglycans (GAGs), they show high size, mean molecular weight (Mw) 50 kDa and heterogeneous chains [7] too great to be fully characterized as they are. Therefore, they must be partially/fully depolymerized by enzymatic or chemical methods. Lyase enzymes generally cleave hexosamine–uronic acid bonds and the final products of their exhaustive digestion are disaccharides bearing at the non-reducing end (NRE) a 4,5-unsaturated (Δ) uronic acid. Nitrous acid is also used to cleave hexosamine N-sulfated bonds at pH 1.5 and free hexosamine at pH 4.0 [8] leading to an anhydromannose at the reducing end (RE) of the depolymerized fragments. CS-A and CS-C, bearing a common repeating unit of d-glucuronic acid β-3-d-*N*-acetylgalactosamine β-4 (GlcA β-3-GalNAc β4) differ in sulfation degree of the 4-O and 6-O positions of GalNAc. DS, also known as CS-B, is a variant bearing a number of GlcA units epimerized to l-iduronic acid (l-IdoA), some of them 2-O sulfated (IdoA2S). HS, expressed on cell surface and basement membranes is a linear, highly heterogeneous, acidic GAG, in nature mainly bound to core proteins in proteoglycans. Depending on the core protein, cell types and cellular environment, the HS polysaccharides show an average chain size ranging from 5 up to 50 KDa and a polydispersity of 1.05–1.6 [7]. In the Golgi apparatus of most mammalian cells the HS precursor, constituted by the alternating (1–4) linked β-d-glucuronic acid α-N-Acetyl d-glucosamine (GlcA-GlcNAc) is synthesized. In the following complex biosynthetic steps some GlcA units are epimerized to IdoA and partially 2-O-sulfated (IdoA2S) while some GlcNAc are N-deacetylated, N-sulfated as well as 3-O and/or 6-O-sulfated leading to a plethora of diverse HS chains. Porcine intestinal mucosa is a source of HS usually in a mixture with CS and DS, remaining after the extraction of heparin [9].

To define compositional and structural characteristic of API samples an orthogonal analytical approach, already adopted for detecting potential contaminants in API heparin batches, was applied for this study [10,11].

At first, danaparoid API was characterized using NMR spectroscopy, mass spectrometry, and molecular weight determination. This allowed a general picture to be obtained of the API in which HS was observed to be the most abundant component in danaparoid. With the aim of also investigating the galactosaminoglycan component, a study was carried out. The structural information here obtained was fundamental for the subsequent part, where two paths were followed to go deeper into the characterization work: (1) fractionation of danaparoid by size and (2) isolation of the two GAG families, which are HS and CS/DS. Both groups of fractions were fully characterized as the parent sample, benefitting from the complementary information coming from the applied analytical methods.

## 2. Results

### 2.1. Danaparoid API Samples

Seven danaparoid API batches and their related size separated fractions were submitted for a full characterization by nuclear magnetic resonance experiments, mass spectrometry, and molecular weight determination.

A study in depth of digested products obtained by chondroitinase ABC (ChABC) digestion treatment (Section 2.2) and the pilot study on size exclusion chromatography (SEC) fractions, described in the first part of Section 2.3.3, were conducted only on one sample.

#### 2.1.1. Determination of Molecular Weight Parameters

**Figure 1:**
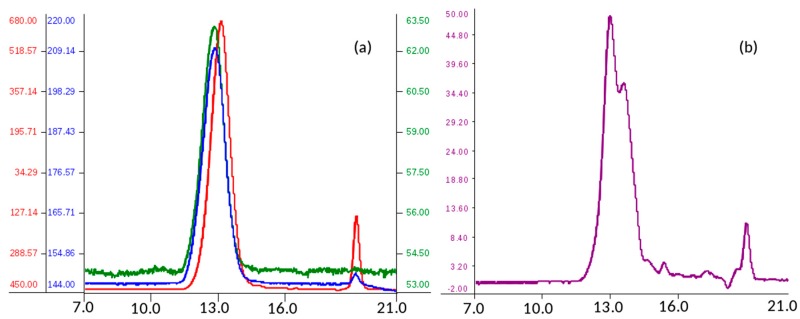
Size-exclusion chromatography profile of a danaparoid sample (CAT272): (**a**) red—refractive index (mV), blue—viscometer (mV) and green—right-angle laser light scattering (mV) detectors; (**b**) UV (mV) detector.

Analysis of molecular weight distribution was performed on seven API samples of danaparoid using high performance size-exclusion chromatography (HP-SEC) on polymeric columns, combined with a triple detector array (TDA) and UV detector. Using a 0.1 M NaNO_3_ aqueous mobile phase, comparable Gaussian profiles exemplified in Figure 1a, were obtained by refractive index, viscometer, and right-angle laser light scattering [12,13].

The software processed data of number average molecular weight (Mn), weight average Mw, and polydispersity (Pd) of analyzed samples were calculated as the mean of two runs. The values of Mn, Mw, and Pd ranging between 3300–3400 Da, 4200–4600 Da, and 1.27–1.36 respectively, indicate for the danaparoid analyzed samples small variation, near to the experimental error (5%).

The non-Gaussian profiles, obtained by UV detection at 260 nm (the eluent suppressed the lower λ signals) (Figure 1b) suggest an inhomogeneous distribution of different components of the complex GAG mixture. This could be ascribed to galactosaminoglycans that are present in high concentration in the higher Mw chains eluted which contain higher molar concentration of UV absorbing N-acetyl groups in comparison to the HS chains of lower Mw and N-acetyl content.

#### 2.1.2. Determination of Sulfate to Carboxylate Ratio

The sulfate-carboxylate ratio, expressing the average disaccharide charge density, was determined in duplicate for all samples by a conductimetric titration method previously reported [14].

The resulting mean ratio of each sample, comprised in the narrow interval 1.19–1.32, indicated a substantial sample equivalence for the sulfation degree, considering the intrinsic variability of the extracted natural GAG components of API danaparoid.

Over the last forty years, the mean ratio values of several analyzed GAG samples, determined in our laboratories with the same method, were found in the range of 1.0–1.2 for CS and DS, 1.0–1.8 for HS and 2.1–2.8 for API porcine mucosal heparin.

#### 2.1.3. Compositional Analysis by 1D and 2D NMR Spectroscopy

**Figure 2:**
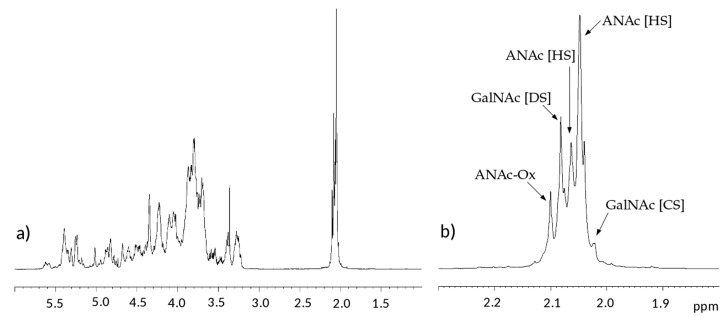
Example of ^1^H-NMR spectrum of a danaparoid sample (CAT272): (**a**) whole spectrum; (**b**) expansion of the acetyl region.

**Figure 3:**
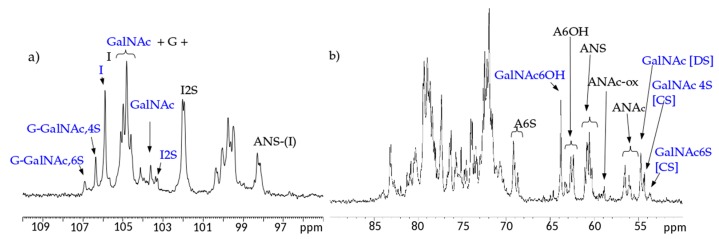
Example of ^13^C-NMR spectrum of a danaparoid sample (CAT272): (**a**) anomeric region; (**b**) ring carbon region. Black: signals attributed to heparan sulfate (HS); Blue signals attributed to chondroitin sulfate (CS) and dermatan sulfate (DS).

Both ^1^H and ^13^C one-dimensional NMR spectra of the samples have similar profiles, in Figure 2 and Figure 3 a proton and a carbon spectra are displayed as examples.

In the proton spectra, the most evident difference is detected in the region of N-acetyl signals at 2.0–2.1 ppm which is correlated to the variable content of galactosaminoglycan components among samples [10,11,15]. Signals of N-acetyl galactosamine of CS are observed at 2.02 ppm, while that of DS is at 2.08 ppm. On the other hand, GlcNAc signals of HS are detected at 2.04 and 2.06 ppm, while those of oxidized N-acetyl hexosamine (ANAc-ox) are at 2.1 ppm (Figure 2b) [16,17,18]. A comparison between acetyl regions of different samples is reported in Figure S1.

**Figure 4:**
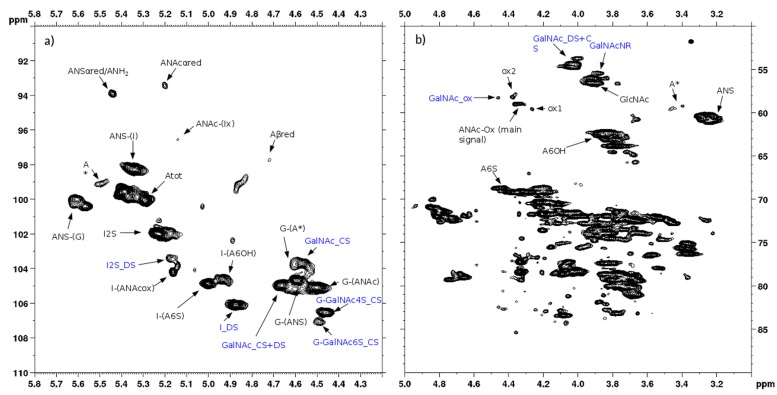
Example of 2D Heteronuclear Single Quantum Coherence (HSQC) NMR of a danaparoid sample (CAT272): (**a**) anomeric region; (**b**) ring region. Black: signals attributed to HS; Blue: signals attributed to DS and CS. Abbreviations in Table S1.

^13^C-NMR spectral analysis allowed signals to be attributed to each danaparoid component (Figure 3) confirmed by HSQC NMR spectra (Figure 4) and comparison with the corresponding literature data [15].

Heteronuclear single quantum coherence spectroscopy (HSQC) 2D NMR experiments on danaparoid samples were recorded by adapting the method recently validated for heparin samples [6].

The qualitative observation of 2D NMR spectra (Figure 4) allows the observation that the majority of peaks can be attributed to the HS component. In the ring region different signals correspond to glucosamines with various sulfation and acetylation substitution (such as ANS, A2*, ANAc, A6OH, and A6S, abbreviations in Table S1); moreover, the signals corresponding to N-acetylated galactosamines and their NRE are present and shown as GalNAc and GalNAc_NR.

In the area 4.6–4.2/57–61 ppm peaks associated to H2/C2 of N-acetyl hexosamine oxidized at the RE due to a bleaching process are observed. The assignments of this group of signals are done based on both literature [17,18,19] and experimental data: the cross-peak at 4.47/58.3 ppm is attributed to the oxidized N-acetylgalactosamines of CS/DS component (GalNAc-ox), whereas the signal at 4.37/58.9 ppm, shown as ANAc-ox, is assigned to oxidized reducing end (N-acetylglucosaminic acid) as described in [16,17]. The other peaks in the same area, displayed as ox1 and ox2, are associated with HS component because they are absent in the enriched CS/DS fractions described in Section 2.4. The hypothesis is that they can be associated to similar oxidized reducing ends on different oligosaccharidic sequences.

In the anomeric region, together with the HS signals, peaks related to DS and CS are displayed (I_DS, I2S_DS, G-GalNAc,4S and G-GalNAc,6S): the uronic acids of both components are useful for the characterization of the API samples.

The assignment of the peak at 5.18/104.2 ppm, previously reported [18], is confirmed as H1/C1 α-l-iduronic acid linked to oxidized N-acetylglucosamine (abbreviation I-(ANAcox)) by 2D NMR experiments.

The integration of characteristic signals of each component allows every contribution to be calculated and produces a detailed characterization of HS in a sample as shown in Table 1 and Table 2.

For the analyzed samples, HS, DS, and CS the percentage ranged from 77.6–88.4%, from 9.0–16.8% and from 2.3–8.0%, respectively. The present NMR quantification was useful for the comparability of API batches. The comparability of the NMR method with the EU Pharmacopeia enzymatic method is ongoing. The NMR method should be not compared equally to the EU Pharmacopeia enzymatic method.

The composition of HS in different danaparoid batches was found very similar, supporting the validity of the process used to extract and purify the product. Moreover, the low percentage of 6-O-sulfation, agrees with the typical composition of an HS structure [20]. The sulfation degree, calculated from the NMR data, confirm the similarity of the HS composition. Values are in the range between 1.62 and 1.70 with an average value of 1.65. The IdoA/IdoA2S ratio of DS and CS-A/CS-C ratio is also similar to that of galactosaminoglycans extracted from porcine mucosa (Table 3) [21].

#### 2.1.4. Chain Mapping by HPLC/ESI MS

An optimized LC/MS method allowed fingerprints of all the samples to be recorded and compared. The ion pair reversed phase high performance liquid chromatography (IPRP-HPLC) coupled to electrospray ionization mass spectroscopic (ESI-TOF MS) detection achieved partial separation of chain components and first level structural assignment. The presence in danaparoid GAGs of epimeric hexosamines required an optimization of the method successfully applied for chain mapping of LMWHs [22,23].

Dibutylamine, the ion pair in the mobile phase, interacting with sulfate groups allowed a better peak resolution when an initial isocratic mode was followed by a linear gradient elution (Figure S2). Good repeatability in three consecutive days and inter-day precision of LC/MS chromatograms was obtained and shown in Figure S3.

**Figure 5:**
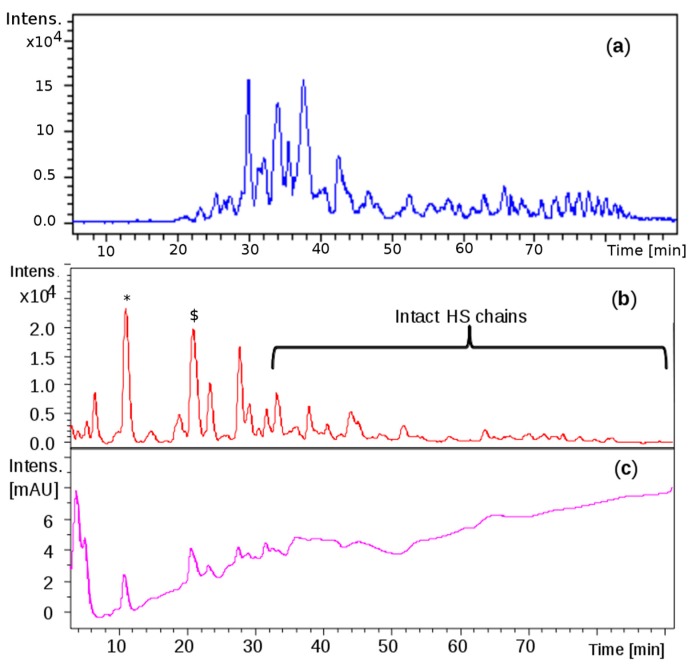
Liquid Chromatography-Mass Spectrometry (LC-MS) profiles: (**a**) one danaparoid sample CAT272; (**b**) ChABC danaparoid digestion product CAT469; (**c**) UV chromatogram at 232 nm of CAT469.

Comparable profiles were obtained for all the samples showing common oligosaccharidic peaks ranging from 1200 up to 7000 Da. Complete base peak chromatogram of sample CAT272 was shown in Figure 5a: minor fluctuations were observed for some samples in the chromatogram portions at 30–40 min and at 75–85 min.

Only the most representative mass signals were attributed to the HS structures: several even and odd chains having 16 additional mass units than the regular oligosaccharidic sequences were identified and, supported by the previous NMR data that highlighted the presence of oxidized residues at the reducing ends, identified as 1-carboxy N-acetyl hexosamine [17,18].

### 2.2. Structural Study of One Danaparoid Sample

Compared to the natural porcine HS, the major component of danaparoid showed some structural modifications that could occur on galactosaminoglycan minor components of danaparoid and need to be further investigated.

One API sample was submitted to digestion with ChABC, the digest was analyzed by ^1^H-NMR and its main signals were assigned to HS sequences. Comparing the N-acetyl region signals with those of the starting material a reduction of DS contribution (Figure S4) became evident; while at 5.9–6.0 ppm the signals of 4,5- unsaturated (Δ) uronic acids at the NRE produced by lyase were observed.

The same mixture was analyzed by LC-MS: the profile showed that the first eluted species (0 to 30 min) generated by the depolymerization of CS/DS could be detected at 232 nm for the presence of 4,5- unsaturated uronic acid due to the lyase action, followed by intact HS species detectable only by MS (Figure 5b,c).

**Figure 6:**
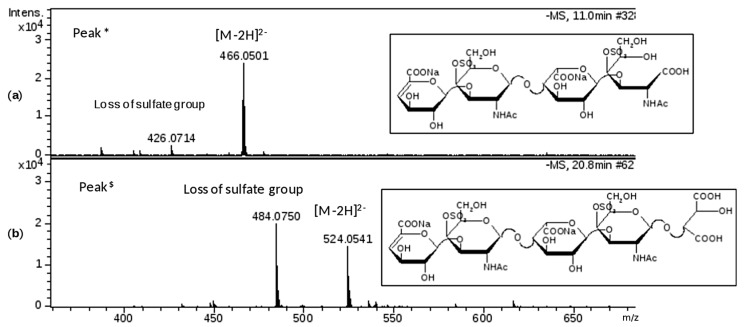
Mass spectra of chromatographic peaks * and ^$^, respectively (as labelled in Figure 5b) and corresponding to structures identified by MS/MS fragmentation experiment (data shown in supplementary material Figures S5 and S6): (**a**) fragment at *m*/*z* 466.0501 (z-2; M 934) attributed to ∆U4,2,2(T1); (**b**) fragment at *m*/*z* 524.0541 (z-2; M 1050) attributed to ∆U5,2,2(Ra). The substitution pattern of DS was used.

This phase was focused on CS/DS oligosaccharides and among them two species with mass value of 934 (*m*/*z* 466 (z-2) RT 10’) and 1050 (*m*/*z* 524.0 (z-2) RT 20’) were detected (Figure 5b) and submitted to MS/MS fragmentation. Two species were identified: one as an unsaturated disulfated, di-*N*-acetyl tetrasaccharide bearing at the RE an oxidized N-acetyl galactosamine residue (ΔU4,2,2(T1)) and an unsaturated disulfated, di-*N*-acetyl pentasaccharide with a remnant (Ra) at the RE (ΔU5,2,2(Ra)) (Figure 6).

The MS/MS spectra show the fragmentation pattern obtained by collision induced dissociation (CID) and structure annotation of numerous product ions allowed to elucidate the structure of the unknown parent ion (Figures S5 and S6). In particular, the characteristic ion product at *m*/*z* 316.0 (z-1) in the MS/MS spectrum of the first tetrasaccharide (M 934), revealed the oxidized galactosamine residue (ANAc,S +O labelled T1) which differs by 16 mass units from its regular form, as similarly observed for HS in Section 2.1.4. While the CID product ion at *m*/*z* 352.1 (z-1) generated from the second oligosaccharide (M 1050) resulted in being particularly informative for the confirmation of Ra terminal (ANAc + C_4_H_4_O_5_).

Considering the whole profile, the main peaks were integrated and their assignments underlined such that the expected final product of the enzymatic cleavage ΔU2,1,1 was accompanied by the variant bearing a remnant Ra at the reducing end ΔU3,2,1(Ra). Other peaks were assigned to sequences having modified terminals T1 or Ra such as the oligosaccharides ΔU6,2,3(T1) and ΔU7,2,3(Ra), but also odd regular species such as the hexosamine A1,1,1, and trisaccharides A3,2,2 and A3,3,2 as well as the pentasaccharide ΔU5,2,2 bearing a uronic acid at the RE.

Since the digestion products detected in the mixture highlighted the presence of oligomers longer than the expected disaccharides, the conditions for an exhaustive depolymerization with ChABC were verified using a higher quantity of enzyme and repeating the reaction on the same sample, without improvement as checked by NMR. Therefore, the enzymatic efficiency was likely decreased by the presence of oxidized and remnant residues at the RE.

This study allowed the presence of unusual reducing ends on CS/DS component to be highlighted: the experimental data agreed in that T1 modification was observed on the galactosamine at the reducing end, while the terminal Ra occurred on uronic acid at the RE.

### 2.3. Preparative Size Exclusion Chromatography of API Danaparoid Samples

**Figure 7:**
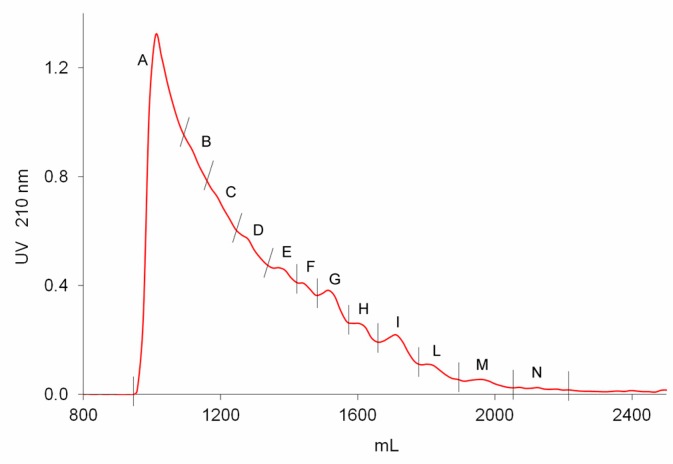
Preparative size exclusion chromatography (SEC) fractionation chromatographic UV 210 nm profile of a danaparoid (CAT277).

Seven batches of danaparoid (CAT271-277) were submitted to preparative gel filtration (SEC) on a Biogel P6 column. Comparable UV curves were detected for all the samples, an example with the indication of the collected fractions is displayed in Figure 7: the molecular weight decreases from A to N.

#### 2.3.1. Determination of the Mw Distribution of SEC Fractions

The Mw distribution of fractions A, B, and C of all the seven API samples was determined and the results, reported in Table 4, showed comparable ranges of weight average Mw and polydispersity. The fraction A, constituted by the highest Mw components, was at the limit of the Biogel P6 chromatographic separation efficiency.

**Figure 8:**
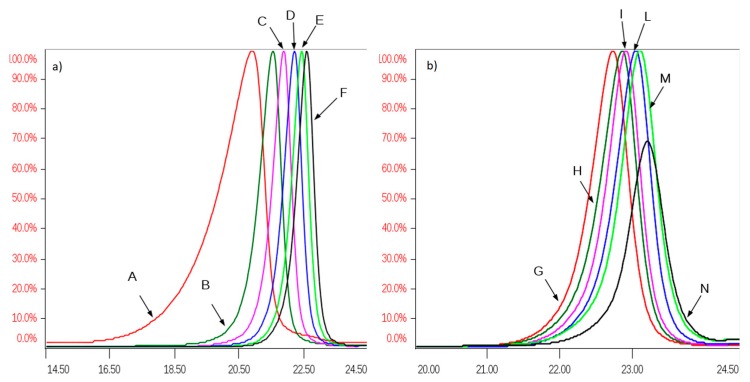
Overlapping of normalized Refractive Index (RI) profiles of SEC fractions of a danaparoid sample (CAT277): (**a**) fractions A–F, range volume 14–24.5 mL; (**b**) fractions G–N, range volume 20–24.5 mL.

All SEC fractions (A–N) obtained from one danaparoid sample (CAT277) were analyzed for the determination of molecular weight parameters (Table 5): components of the subsequent fractions showed decreasing Mw and very low Pd values; the partial overlapping between adjacent fractions was displayed by their Refractive Index (RI) profiles in Figure 8.

#### 2.3.2. 2D-NMR Spectroscopic Analysis of SEC Fractions of Danaparoid Samples

The 2D-NMR spectra of the danaparoid SEC fractions, considering some signals characteristic for each component, allowed it to be underlined how the proportion of GAG species varies over fractions.

Inspection of the H2/C2 region displays that the galactosamine (CS/DS) signals had more or equal intensity than those of glucosamine (HS) in the high Mw oligomers corresponding to fractions A and B followed by a progressive decrease in the subsequent fractions up to being absent in fractions L, M, and N.

The signal of C2/H2 of NS,3S,6S glucosamine (A2*) is present with variable intensity in almost all fractions, up to being undetected or in traces in fractions A and B.

The peaks related to oxidized RE residues at 4.6–4.2/57–61 ppm were variably present in almost all fractions.

The comparison between fractions obtained from different APIs was performed by analyzing the anomeric regions of the HSQC spectra (Figure S7), considering the peculiar disaccharides, IdoA2S-GlcN for HS, IdoA-GalNAc for DS and GlcA-GalNAc 4 and/or 6 sulfated for CS.

By visual inspection the highest content of CS and DS components was found in fraction A, the intensity of their cross-peaks being superior to those of IdoA2S of HS, then decreasing gradually in all the danaparoid samples, while the HS oligosaccharides increased upwards to become the sole components in fractions L to N. The DS components seemed to be more abundant than those of CS which disappeared early, as the signals of GlcA linked to GalN6S in comparison with those of GlcA linked to GalN4S. Variation of the CS/DS component content and distribution was qualitatively evaluated in HSQC spectra and summarized as scores in Table 6, resulting in being slightly different among the seven API danaparoid samples. This is considered normal for a natural extraction product.

#### 2.3.3. Sequencing of Danaparoid SEC Fractions by LC/MS Analysis

A pilot study by the IPRP-HPLC/MS method was addressed to sequence the SEC fractions of a danaparoid sample (CAT277) (Figure 7) to achieve a first level structural assignment of components and identify probable modified structures on shorter chains. This explorative study was conducted by analyzing a restricted portion of each SEC fraction of the danaparoid sample, with the aim of simplifying the expected complexity of sequences and eventually identifying structure modifications. These selective analyses provided only a partial picture of the whole fractions but also the detection of minor modified sequences useful for the study.

**Figure 9:**
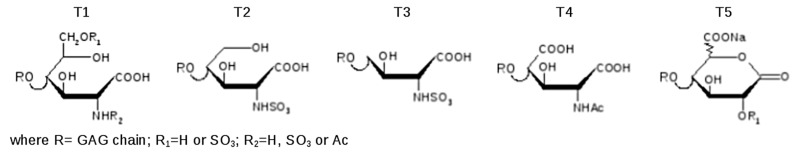
Structures of oxidized Reducing End (RE) residues compatible with the observed *m*/*z* values (the substitution pattern was in accordance with the observed species listed in Table 7).

This approach led to the identification in the fractions E to N of few HS regular sequences and a majority of HS chains bearing oxidized residues at the reducing end, likely 1-carboxylated glucosamines, shown as T1 in Figure 9 [17,18,19] applying the code already used for the similar modification on CS/DS (Section 2.2). Less abundant modifications were observed and displayed as T2, T3, T4 and T5 (Figure 9).

**Figure 10:**
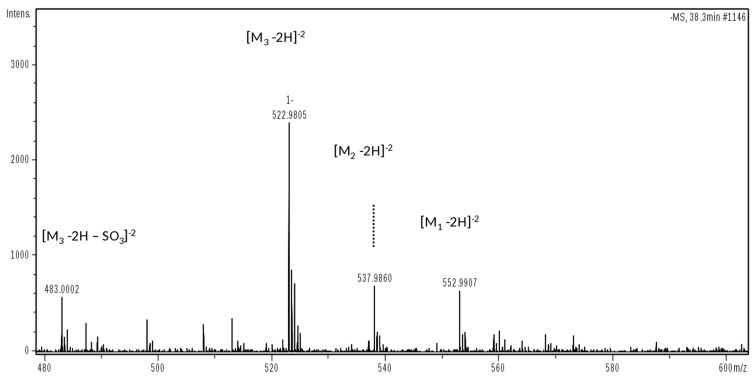
Mass spectrum displaying the different oxidation forms of oligosaccharide U4,5,0 from M_1_ to M_3_ derivative: *m*/*z* 552.9907 (z-2, M_1_ 1108) identified as U4,5,0 (T1); *m*/*z* 537.9860 (z-2, M_2_ 1078) identified as U4,5,0 (T2); *m*/*z* 522.9805 (z-2, M_3_ 1048) identified as U4,5,0 (T3).

In particular, the species exhibiting T2 and T3 end residues, detected in a minor peak of fraction N, can be explained by further oxidation of the secondary alcohol and decarboxylation steps of terminal T1 (Figure 10). Confirmation of the molecular formula is supported by a very low error (<10 ppm) between experimental and theoretical mass values.

Concerning the T4 modification, observed in fraction H, only a high resolution MS measurement allowed this oxidized residue to be discriminated from a regular structure.

**Figure 11:**
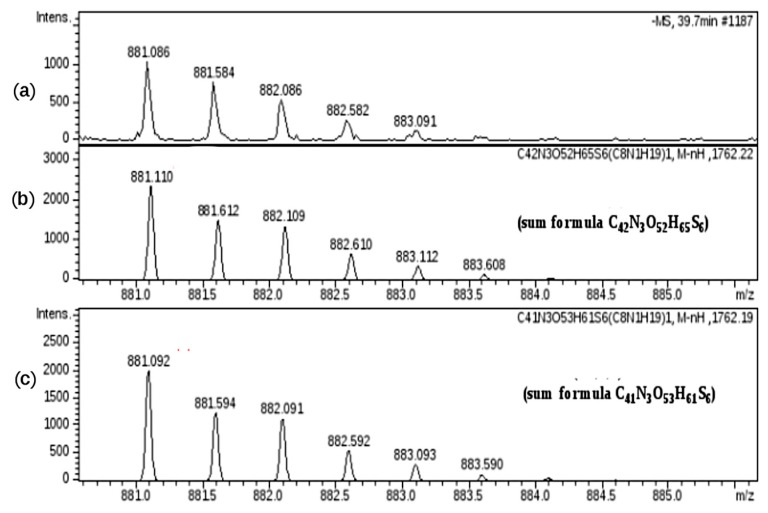
Mass signal at *m*/*z* 881.086 (z-2): (**a**) experimental *m*/*z* and isotopic distribution; (**b**) theoretical *m*/*z* and isotopic distribution corresponding to the regular structure of U6,6,3 (sum formula C_42_N_3_O_52_H_65_S_6_); (**c**) theoretical *m*/*z* and isotopic distribution corresponding to the oxidized structure U6,6,3(T4) (sum formula C_41_N_3_O_53_H_61_S_6_).

The experimental mass signal at *m*/*z* 881.086 (z-2), shown in Figure 11, at first sight seemed to correspond to the regular hexasaccharide U6,6,3 (sum formula C_42_N_3_O_52_H_65_S_6_). A more detailed mass investigation, revealing a high mass error between experimental and theoretical values (22 ppm), suggested another possible structure modification as U6,6,3(T4) corresponding to a sum formula of C_41_N_3_O_53_H_61_S_6,_ showing a good overlap of mass signals with a decrease of the error to 2 ppm.

In fractions F, H, and L, some mass values differing by two Daltons (-2H) with respect to the HS regular sequences were observed. The hypothesis was that after the oxidation of carbon 1 a water loss occurred to give the lactone (T5 in Figure 9) and lacking 2 Da. The mass accuracy of both T1 and T5 modifications found in fraction F was verified also by ESI-FT MS: the error on theoretical mass was minor or equal to 1.5 ppm.

After the pilot study the same analytical method was applied to the SEC fractions isolated from seven API samples. A qualitative observation of the LC profiles of the corresponding fractions showed a good similarity mainly among fractions D to N, containing LMW chains. A higher variability was observed among the first eluted fractions (A to C), containing mainly CS/DS high Mw chains endowed with an intrinsic heterogeneity of natural extractive GAGs such as danaparoid and whose content varied among API samples. A compositional analysis was performed integrating the main peaks where the principal detected species were investigated: the structures were the same inside each fraction group, while different oligomers were observed in different fractions.

**Figure 12:**
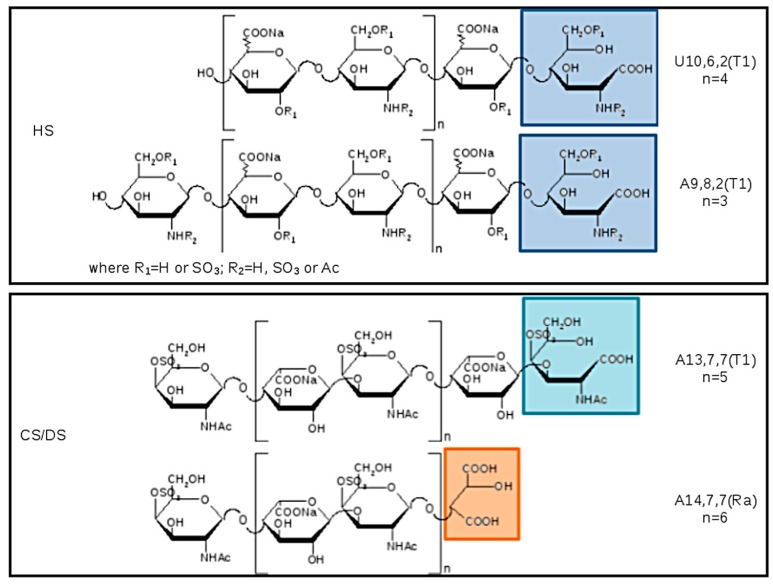
Example of structures detected in SEC fractions, the modified RE are highlighted in the boxes: HS sequences are displayed in the upper panel with the terminal T1 at the RE and with the uronic acid or the glucosamine at the non reducing end (NRE) in even or odd oligomers, respectively. CS/DS structures are shown in the lower panel (the substitution pattern of DS is used as example because it is more abundant than CS): two possibly reducing ends are T1 and Ra, the galactosamine is placed at the NRE.

The list of main species found in SEC fractions is shown in Table 7, together with the GAG assignment and eventual additional species previously identified in the pilot study. Components are indicated by: A for hexosamines or U for uronic acids as NRE residues, followed by three numbers referring respectively to oligosaccharidic units, sulfate, and N-acetyl groups. The number of oligosaccharidic units includes both RE and/or NRE variants (T1–T5 and Ra), examples of sequences are shown in Figure 12. The assignment of the *m*/*z* value to a single GAG component was based on the knowledge of their N-acetylated, N-, O-sulfated degrees.

The main components of fractions A–C were assigned to CS/DS, and those of fractions D to N to HS components. Some unassigned mass values were present, but detected in all samples.

Almost all the detected species showed a structural modification of the reducing end residue. Oxidized hexosamine T1 was present in both CS/DS and HS sequences, while the remnant Ra, of oxidized uronic acid, seems to be present only on CS/DS chains.

In principle, oxidation of reducing end residues can occur both on hexosamine and uronic acid [24]. Actually, whereas for odd sequences experimental mass data permitted the certain identification of ending residues thus confirming the presence of T1 on RE hexosamine, for even sequences terminal RE and NRE residues cannot be distinguished, and oxidized residues were not precisely assigned accordingly. Considering the literature data [17,18], in Table 7 and Figure 12 the variant T1 was attributed to the RE hexosamine of even sequences bearing a uronic acid at the NRE.

The terminal T5 (lactonized form of T2) was observed on both even and odd sequences as shown in Figure 9 and Table 7.

### 2.4. Isolation of GAG Components of Danaparoid and Compositional Analysis

Three danaparoid samples were chosen to perform the isolation of HS and CS/DS components, based on their different content of HS-DS-CS that range from 78.0–87.2%, from 9.8–14.0% and from 3.0–8.0%, respectively (NMR data).

The parent samples were submitted to complementary procedures to depolymerize selectively the two GAG families of danaparoid: for the isolation of HS the chondroitinase ABC was applied, while the isolation of CS/DS was performed using heparin lyase III followed by a chemical reaction using nitrous acid.

The depolymerization methods, described in Section 5, led to the isolation of three enriched fractions of HS and CS/DS. The weight recovery for the enriched HS fractions was about 78–81%, while those of CS/DS range from 15–26%.

Both components were fully characterized by NMR, molecular weight distribution, LC-MS chain mapping and only for HS fractions by LC-MS disaccharidic analysis as subsequently reported herein.

#### 2.4.1. Molecular Weight Data

Molecular weight distribution was determined by triple detector array (TDA) for both families of samples: the enriched CS/DS fractions range from 6200 to 7100 Da, while for those enriched HS components the Mw varies from 3300 to 3500 Da and is consistent with the data of the SEC fractions. The polydispersity is lower for the HS species (1.13) than for the CS/DS ones (1.19), both are lower than the API samples.

**Figure 13:**
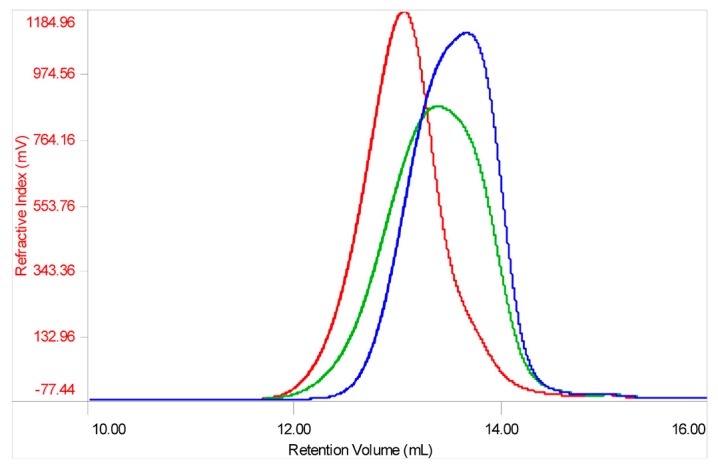
Refractive Index profile overlay of a danaparoid sample (CAT272 in green), its enriched CS/DS (red) and HS (blue) fractions.

In Figure 13 The Refractive Index (RI) profile overlay of a parent API sample and its enriched HS and CS/DS fractions displays the presence of CS/DS components in the higher Mw with respect to the API, as already observed in the NMR and MS data regarding the SEC study. On the other hand, the enriched HS fraction is observed in the mid–lower Mw range.

#### 2.4.2. NMR Observations

**Figure 14:**
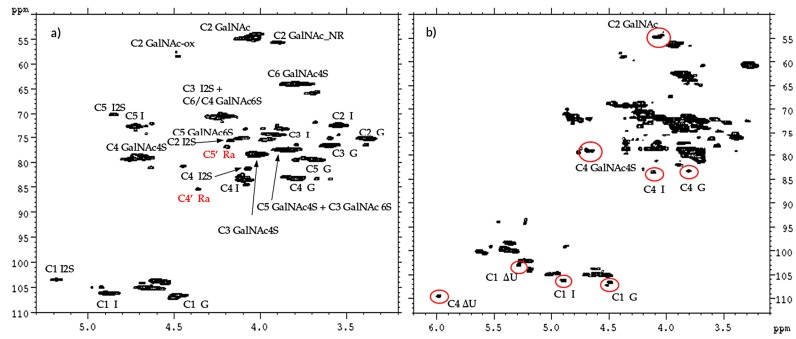
HSQC NMR spectra of CS/DS (**a**) and HS (**b**) fraction of danaparoid CAT272. Signals belonging to CS/DS are circled in red.

The 2D-NMR-HSQC assignments of the CS/DS component were done according to literature data [19,25,26] as shown in the spectra of one CS/DS enriched fraction (Figure 14a): the chemical shifts of 4.36/85.4 ppm and 4.19/76.8 ppm, in red, were in agreement with the positions C4’ and C5’ of remnant Ra. While the CS/DS fraction does not contain signals of HS, the spectrum of the HS fraction also shows signals typical of CS/DS oligomers, highlighted in red in Figure 14b.

**Figure 15:**
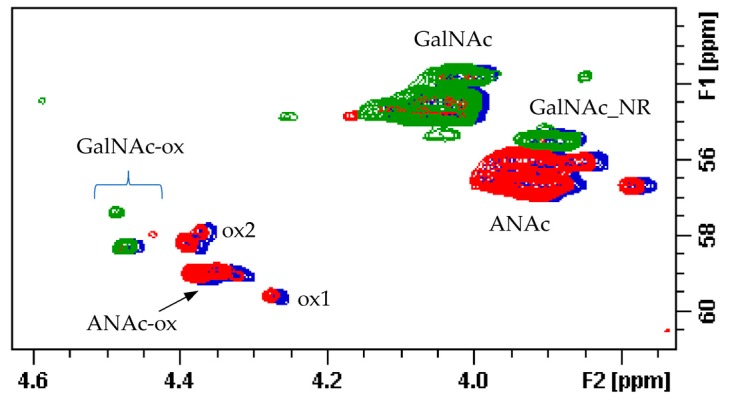
Superimposition of HSQC spectra (C2 region) of a danaparoid sample (CAT272 in blue), its enriched HS (red) and CS/DS fractions (green).

The superimposition of the HSQC spectra of HS and CS/DS fractions with that of the danaparoid starting material allows some peaks to be assigned correctly: signal at 3.90/55.5 ppm belongs to the C2 of the NRE GalNAc residue of CS/DS [25]. The signals in the oxidized region at 4.39/58.1 ppm and 4.27/59.5 ppm (shown as ox1 and ox2 in Section 2.1) belong to HS, being absent in the spectrum of the CS/DS fraction (Figure 15).

The composition of HS and CS/DS in the corresponding enriched fractions is very similar to that found in the parent danaparoid samples, demonstrating the robustness of the methods of separation.

The enriched CS/DS fractions, free from HS, display DS as the most abundant species ranging from 63–76%. On the contrary, the enriched HS fractions include a part of CS/DS that varies between 5% and 14%.

#### 2.4.3. LC-MS Chain Mapping Analysis of CS/DS Fractions

The LC-MS analysis of enriched CS/DS fractions show the simplification of the region of short chains (RT 20–40 min) due to the removal of the HS component by the analytical depolymerization/purification procedure, and contemporarily the enrichment of CS/DS species in the region of long chains (RT 50–90 min).

**Figure 16:**
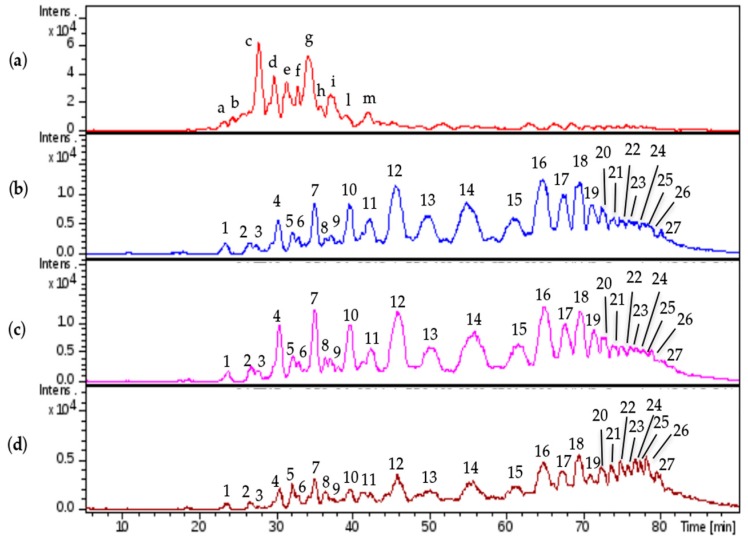
LC-MS profiles comparison: (**a**) danaparoid sample CAT272; (**b**) CS/DS isolated from CAT271; (**c**) CS/DS isolated from CAT272; (**d**) CS/DS isolated from CAT275.

The investigation in depth of mass spectra at the apex of each peak allows the main mass signals (Figure 16) to be assigned: CS/DS chains from A6,2,3 to A30,15,15 oligomers were identified (Table S2), almost all of them having both the oxidized variants T1 and Ra remnant due to the production process. These results are in agreement with the data obtained by SEC fractions in which the CS/DS sequences from A13,7,7 to A27,14,14 were identified. The isolation of the CS/DS component allowed their shorter oligomers previously masked by the HS component in the API danaparoid sample to be detected.

The qualitative comparison of LC-MS profiles of the three isolated CS/DS samples underlines a good similarity between the CS/DS fractions isolated from CAT272 and CAT271, while for those isolated from CAT275 the lower molecular weight species (from 20 to 60 min) are less represented with respect to the higher Mw ones eluting from 60 to 85 min in comparison with the other two samples. This observation is in agreement with SEC data in which the variation of CS/DS content is displayed as scores in Table 6 and for CAT275 their reduction is observed in longer fractions than for CAT271 and CAT272.

#### 2.4.4. LC-MS Chain Mapping Analysis of HS Enriched Fractions

**Figure 17:**
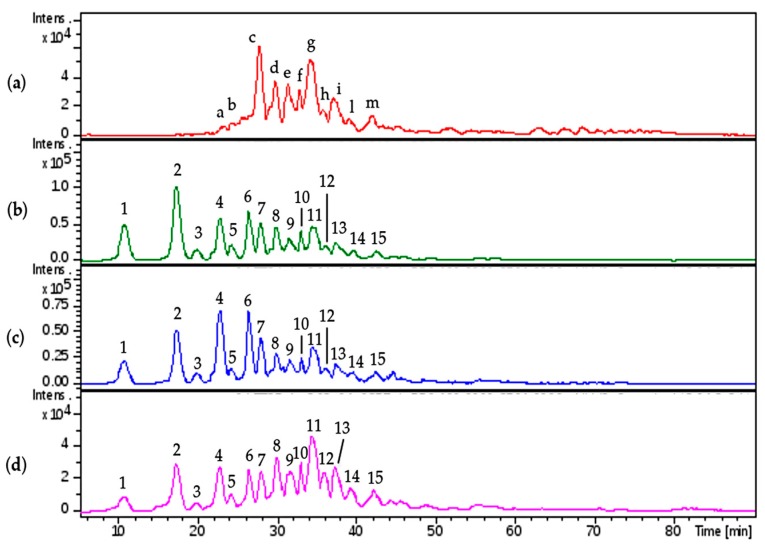
LC-MS profiles comparison: (**a**) danaparoid sample CAT272; (**b**) HS isolated from CAT271; (**c**) HS isolated from CAT272; (**d**) HS isolated from CAT275.

The enriched HS fractions were also analyzed by LC-MS and compared with danaparoid (Figure 17).

The assignments were reported in accordance with the retention time, the majority of the peaks (n.7–15) were attributed to HS oligomers with the modification T1. Regular sequences of HS were observed with less intensity assigned to A5,6,0; A7,6,1; A7,5,0.

The comparison of the region from 30 to 45 min for the three isolated samples displayed slight differences in the intensity of the peaks.

The comparison of isolated HS and danaparoid (Table S3) allowed the correspondence of HS sequences in the isolated HS (peaks n.7–15) and in danaparoid (peaks from ‘c’ to ‘m’) to be observed; in the range of shorter species (peaks n.1–6 and at 29.1 min) there were pentasaccharides with 3 acetyl and hexa- and hepta-saccharides with a double bond on uronic acid, not present in the parent danaparoid that should be related to the residual presence of CS/DS fragments from the enzymatic process and not removed by purification steps (as already observed in NMR spectra).

To complete the compositional analysis, the HS enriched fractions, along with a nadroparin and a heparin samples were submitted to enzymatic digestion using a mixture of heparinase I, II, III.

**Figure 18:**
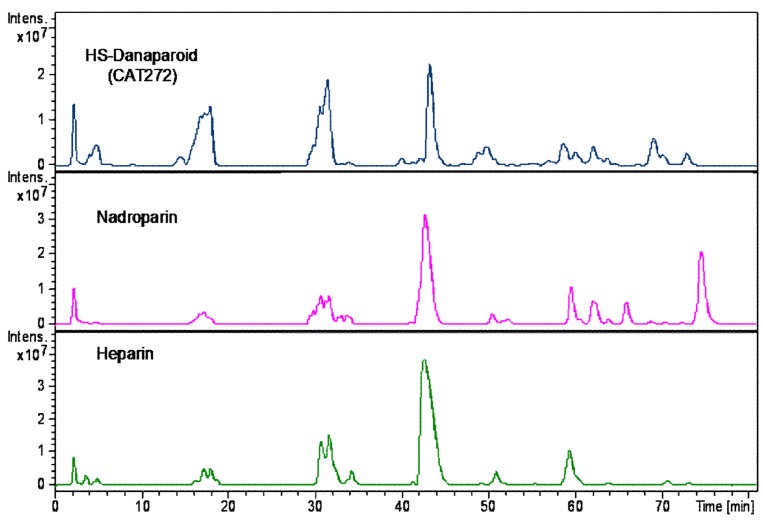
LC-MS profiles of HS danaparoid (CAT272), nadroparin and heparin digested by heparinases I, II, III.

A qualitative inspection of the profiles highlighted a comparable oligosaccharidic composition among the digestion products of all the enriched HS fractions, some differences were observed in the nadroparin and heparin digest components (Figure 18).

The peaks identified by comparison with commercial standard disaccharides, were detected in all digested samples. As regards the species obtained from the enriched HS fractions regular unsaturated oligomers were observed together with some saturated odd and even sequences. Confirming the previously described data, some tetrasaccharides with the variant T1 at RE were present, only a very low peak corresponding to an unsaturated monosulfated, *N*-acetyl disaccharide bearing the T1 at the RE was detected supporting the hypothesis that the variants at RE inhibit the lyases.

An unsaturated tetrasaccharide with one acetyl and four sulfates was observed together with some species, Δ4,3,1, A3,4,0, and A3,5,0 that should be related to the sequence AGA*IA not cleaved by lyases [27,28].

Based on the ratio between N-acetylated and N-sulfated disaccharides, calculated by the commercial standard disaccharides peaks, the enriched HS fractions were compared with nadroparin and heparin. The data shown as percent in Table 8 allow the substantial different nature of heparin to be underlined in respect of the glucosaminoglycan component of danaparoid that has an HS structure [29,30]. This semi-quantitative analysis of N-acetylated/N-sulfated disaccharides ratio was performed on extracted ion chromatograms (EIC) of mass signals corresponding to mass values of disaccharides (the sum of ∆U-ANAc, ∆U-ANAc6S, ∆U2S-ANAc, ∆U2S-ANAc6S with respect to the sum of ∆U-ANS, ∆U-ANS6S, ∆U2S-ANS, ∆U2S-ANS6S percent).

## 3. Discussion

The danaparoid characterization was focused on a compositional study of intact API samples in obtaining their general picture, followed by the study of fractions differing in size and of their GAG components. Both APIs and their fractions were analyzed with complementary techniques following an orthogonal analytical approach.

The analyzed API samples resulted in similar data for every applied technique such as NMR spectra, LC-MS profiles, and HP-SEC-TDA curves; their variability was included in the natural variation typical of extractive products.

Danaparoid was a low-molecular-weight mixture of HS, CS, and DS, confirmed by experimental weight average molecular weight (Mw) ranging from 4200 to 4600 Da. The charge density determined as sulfate-to-carboxylate ratio was between 1.19 and 1.32.

By analyzing the NMR spectra, it was confirmed that the main species is an HS glucosaminoglycan, based on the sulfation/acetylation pattern. Dermatan sulfate and chondroitin sulfate were present quantitatively as secondary and tertiary components, respectively. Among the peaks relative to regular mono- or disaccharides of GAGs, some signals connected to the bleaching process were detected and attributed to oxidized gluco-/galactosamine at the reducing end (shown as T1). This modification, corresponding to the addition of one oxygen to the mass values of regular sequences, was confirmed by mass spectrometry both on HS and CS/DS. During the in-depth study on the galactosaminoglycan component, the remnant 3-O tartaric acid (Ra) at the RE was also detected.

The size fractionation was performed for all danaparoid batches and their fractions were submitted for further studies. NMR spectra were evaluated by means of the main characteristic signals of each component of danaparoid, through the variation of their anomeric peaks in every fraction and then among the starting batches: for all APIs the highest quantity of CS/DS component was found in the higher molecular weight fraction A, then decreasing down to fractions L, M, and N containing only HS. The different CS/DS content as well as their different distribution could be explained by variation in the starting natural sources. The presence of HS component in all fractions was ascertained by NMR.

The MS data of SEC fractions were evaluated qualitatively from the chromatographic profiles. These showed slight variations among batches in the range of longer chains where the major variability was expected. From the compositional point of view, the chain size decreased and sequences varied from fraction A to N, the main species found in the fraction of the same size of different API batches had an analogue composition.

In particular, investigating the main mass values, the fractions A to C showed CS/DS sequences having both T1 and Ra variants at RE; while in the subsequent fractions, structures attributed to HS were identified and characterized not only by the most abundant reducing end variant T1 but also by regular sequences and, less representative but structural interesting, variants T2, T3, T4, and T5. The majority of the HS components of all fractions were found to be odd oligosaccharides with a glucosamine (A) at the NRE; in fractions F and H only even sequences were identified; in the fractions containing shorter chains odd and even sequences were in similar abundance. Odd oligosaccharidic chains were not usually found in extractive GAG sequences even if present in low amounts in the LMWHs, enoxaparin and dalteparin [23] and highly represented in parnaparin [31] and γ-heparin [32] obtained by radical depolymerization.

The Mw range of fraction components, determined by MS is comparable with that obtained by the HP-SEC-TDA method which underlined also the closeness of contiguous fraction components, in particular for F to N included from 3000 to 1900 Da.

The isolation of components starting from three intact API samples was performed applying depolymerization procedures, using the proper enzyme and/or chemical reaction, followed by multiple purification steps. All of them were submitted for full characterization: Mw distribution, NMR, chain mapping, and only for HS fractions disaccharidic analysis by LC-MS.

The isolated CS/DS fractions were pure and the molecular weight analysis confirmed that the galactosaminoglycans were mainly present in the high molecular weight range of danaparoid, having a weight average molecular weight (Mw) from 6200 to 7100 Da. On the other hand, the HS fractions should be considered enriched HS fractions with CS/DS still being present (5–14%), their weight average Mw was found in the range from 3300 to 3500 Da, consistent with SEC fractions data.

Analyzing their NMR spectra, the fractions isolated from the three API samples showed a composition similar to that of the corresponding species present in the parent API samples, supporting the robustness of the separation procedures. Taking advantage of the purity of CS/DS fractions, some NMR signals were correctly assigned to the right component such as the C2/H2 of the NRE GalNAc residue at 3.90/55.5 ppm. In the region of oxidized residues two signals ox1 and ox2, hypothetically belonging to sequences similar to that bearing T1 variant, were attributed to HS because they were not observed in the isolated CS/DS fractions. The content of DS was evaluated by NMR in the isolated CS/DS fractions compared with that of CS and resulted ranging from 63% to 76%.

Moreover, also the LC-MS profiles of isolated CS/DS fractions were simplified by the removal of HS component, in the region of both short and long oligomers. The observed oligomeric size distribution was in good agreement with the SEC data and allowed the detection of higher oligomers (range 1271 (dp6) to 8282 Da (dp30)) bearing the variants T1 or Ra at the RE.

The study of enriched HS fractions allowed the confirmation of components already observed in danaparoid (dp5 to dp8) and the disaccharidic analysis, performed in comparison with nadroparin and heparin samples, provided compositional data in accordance with SEC data. Moreover, the evaluation of N-acetyl and N-sulfate ratio underlined that the glucosaminonoglycan component of danaparoid is heparan sulfate differing from those obtained from nadroparin and heparin samples.

## 4. Materials and Methods

### 4.1. Reagents and Starting Materials

Seven danaparoid API batches (CAT271-277), one heparin sodium USP and one Nadroparin calcium samples were provided by Aspen Oss B.V., Oss, Netherlands. Heparin lyases I (EC 4.2.2.7), II and III (EC 4.2.2.8) were purchased from Grampian Enzymes, Aberdeen, UK. Chondroitin ABC lyase from *Proteus vulgaris* (EC 4.2.2.4), ammonium acetate (≥98%), sodium azide (≥99.0%), sodium nitrate (≥99.0%), sodium dihydrogen phosphate monohydrate (>98%), sodium hydrogen phosphate dihydrate (≥99.0%), trimethylsilyl-3-propionic acid (TSP 98% D), dibutylamine (≥99.5%), acetic acid (glacial, 99.9%), acetonitrile (LC-MS grade), methanol (LC-MS grade), ammonium chloride (≥99.5%), sodium nitrite (>95%), sodium tetraborate (≥98%), hydrochloric acid (≥37%) were purchased from Sigma Aldrich (Milan, Italy); calcium acetate (≥97%) from BDH; sodium acetate (≥99%) and NaOH (≥99%) from Merck (Kenilworth, NJ, USA); Amberlite IR 120 H^+^ and 0.1 M NaOH from Fluka Analytical (Milan, Italy).

Ethanol (96%) was purchased from Girelli Alcool (Milan, Italy); ethylenediaminetetraacetic acid (EDTA D16, 98%) from Cambridge Isotope Laboratories (Tewksbury, MA, USA) and deuterium oxide (≥99.9%) from Euriso-top (Saint-Aubin, France). Deionized water (conductivity less than 0.15 µS) was prepared with an osmosis inverse system (Culligan, Milan, Italy).

### 4.2. Molecular Weight Determination

Molecular weight determinations were performed using an HPLC system combined with a Viscotek mod. 305 Triple Detector Array [12,13]. The HPLC Viscotek equipment was made up by a Knauer Smartline 5100 pump, a Biotech Degasser model 2003, and an HTA autosampler model HT310L. The detector system was composed of right angle laser light scattering (90° angle geometry), refractive index and viscometer; all detectors and the separation columns were contained in an oven compartment.

Two chromatographic conditions were applied: (I) for SEC fractions (Section 2.3.1) two silica columns G3000SWXL+G4000SWXL TSK GEL (7.8 mm ID × 30 cm, Tosoh Bioscience S.r.l., Rivoli, Torino, Italy) preceded by precolumn TSK GEL SWXL GUARD (7 µm, 6.0 × 40 mm, Tosoh Bioscience) were used eluting with ammonium acetate 0.1 M, sodium azide 0.02% at 0.6 mL/min ± 10% setting up the temperature at 30 °C; (II) for API samples (Section 2.1.1) and isolated fractions CS/DS and HS (Section 2.4.1) the chromatographic elution was performed at 0.6 mL/min ± 10% with sodium nitrate 0.1 M, sodium azide 0.05% on two polymeric columns G3000PWXL+G2500PWXL TSK GEL (7.8 mm ID × 30 cm, Tosoh Bioscience) at 40 °C.

The samples were dissolved with a concentration between 8 and 12 mg/mL in the mobile phase used for the elution; 100 μL of each solution was injected. All chromatographic systems were calibrated using for (I) a pullulan (PSS, Mainz, Germany) and for (II) a polyethylene oxide (Agilent Technologies, Santa Clara, CS, USA), both are certified standards of known Mw, polydispersity and intrinsic viscosity. The data elaboration was performed with OmniSEC software, version 4.6.2 (Malvern, UK).

### 4.3. Sulfation Degree Determination

The sulfation degree of all samples, expressed as sulfate to carboxylate molar ratio, was determined in duplicate by conductimetric titration following the method proposed by Casu et al. [14]. A sample of ~75 mg of the acidic form of danaparoid, previously exchanged on Amberlite IR 120 H+, in 100 mL of distilled water was titrated with 0.1 M NaOH using an automatic titrator (Titrando 888, Metrohm Italiana S.r.l., Origgio, Varese, Italy) equipped with a conductivity cell (constant = 0.76 cm^−1^).

### 4.4. NMR

About 250 mg of API batches were dissolved in 2.5 mL of D_2_O, and analyzed by using a Bruker AV 500 MHz (125 MHz for ^13^C) NMR spectrometer (Karlsruhe, Germany). The carbon spectra were recorded with a pulse delay of 1 s and 20 k scans.

About 35 mg of danaparoid samples were dissolved in 0.6 mL of buffer solution pH 7.1 (buffer phosphate 0.15 M, EDTA-D16 0.3 mM) in D_2_O with 0.12 mM TSP; while the fractions isolated (20 or 35 mg) during the experimental activity were dissolved in 0.6 mL of D_2_O. Both groups of samples were analyzed by using a Bruker Avance III HD NMR spectrometer operating at 500 MHz (^1^H) equipped with TCI cryoprobe (Karlsruhe, Germany). The proton spectra were acquired with presaturation of residual HDO signal, 16 scans and 12 s of pulse delay. The Heteronuclear Single Quantum Coherence (HSQC) spectra were recorded with the library Bruker pulse sequence hsqcetgpsisp2.2, a pulse delay of 2–5 s and 8–24 scans. The spectra elaboration was done using Bruker TopSpin software, version 3.2 (Karlsruhe, Germany).

### 4.5. LC-MS Analysis

LC-MS analysis of all of samples was performed using ion-pair reversed-phase separation on a Kinetex-C18 column (2.1 mm × 100 mm, ODS 2.6 μm, 100 Å, Phenomenex, Aschaffenburg, Germany) with pre-column filter (Phenomenex, Aschaffenburg, Germany) at room temperature coupled with mass spectrometer. The injected volume was 5 μL at a concentration of about 5 mg/mL.

A binary solvent system was used for a multi-step gradient elution using solvent A (dibutylamine 10 mM, acetic acid 10 mM in water) and solvent B (dibutylamine 10 mM, acetic acid 10 mM in acetonitrile) with the following schedule: (I) for SEC fractions of pilot study (first part of Section 2.3.3) at flow rate of 0.1 mL/min, 15% B for 5 min, linear gradient from 15% to 26% B in 25 min, 26% B for 20 min, linear gradient from 26% to 40% B in 35 min, fast linear gradient from 40% to 60% in 13 min, hold 60% B for 10 min, then return to 15% B in 2 min and hold for 30 min 15% B; (II) for API batches, isolated fractions of CS/DS and HS and all SEC fractions at flow rate of 0.15 mL/min, 15% B for 5 min, linear gradient from 15% to 24% B in 22 min, 24% B for 23 min, linear gradient from 24% to 40% B in 50 min, fast linear gradient from 40% to 60% B for 3 min, then 60% B for 10 min, then return to 15% B in 2 min and hold for 20 min at 15% B.

The samples were analyzed on an Ultimate 3000 HPLC-UV system (Dionex, Sunnyvale, CA, USA) coupled to an ESI-Q-TOFMS MicrOTOF-Q (Bruker Daltonics, Bremen, Germany). The mass spectrometer setting was as follows: ESI in negative ion mode (capillary voltage +3.2 kV); nitrogen, used as nebulizer and heater gas, flowed at 7 L/min, +180 °C and pressure 0.9 bar; mass range of 200–2000 *m*/*z*. Only the SEC fractions F, I–N were analyzed on an HPLC-UV system (Agilent Technologies, Santa Clara, CA, USA) coupled to an ESI FT-ICR MS Solarix (Bruker Daltonics, Bremen, Germany) using the elution schedule (II) and MS conditions were set up in negative polarity with a capillary voltage of +3.2 kV, nitrogen at the flow rate of 3.7 L/min, temperature of +180 °C and pressure of 1 bar, mass range 200–3000 *m*/*z*.

The MS^2^ fragmentation experiments were performed on selected ions isolated in the quadrupole collision cell by a width of 5 Da and activated by collision-induced dissociation (CID) at the collision energy of 25 eV.

LC-MS analyses of enzymatic digested mixture (Section 2.4.4) were performed on an ESI-IT amaZon SL (Bruker Daltonics, Bremen, Germany) using solvent A (dibutylamine 10 mM, acetic acid 10 mM in water) with solvent C (dibutylamine 10 mM, acetic acid 10 mM in methanol) at flow rate of 0.1 mL/min, 10% C for 5 min, linear gradient from 10% to 35% C in 35 min, linear gradient from 35% to 50% C in 45 min, from 50% to 90% in 13 min, then hold at 90% C for 5 min, then return to 10% C in 2 min and hold for 25 min 10% C. The mass spectrometer parameters were as follows: ESI in negative ion mode with a capillary voltage of +3.8 kV, nitrogen at +200 °C, 9 L/min with a nebulizer pressure of 30 psi, mass range 200–1500 *m*/*z*.

### 4.6. Size-Exclusion Chromatography

The fractionation method was applied to seven API samples: about 250 mg dissolved in 2.5 mL of H_2_O were loaded onto two columns in series (5.0 × 90 cm each) of Bio-Gel P6 (BIO-RAD Laboratories S.r.l., Milan, Italy), the elution was performed with 0.25 M ammonium chloride solution at a flow rate of 1.8 mL/min. Tubes of 18 mL were collected, and their absorbance at 210 nm was monitored using a variable-wavelength UV detector (Varian Cary 50scan, Agilent Technologies, Santa Clara, CA, USA). Based on the profile, twelve fractions with decreasing molecular weight were recovered (labelled from A to N).

Two desalting/purification methods were applied and described as follows: (I) one column (5.0 cm × 90 cm) filled with TSK HW40S resin (Tosoh Bioscience S.r.l., Rivoli, Torino, Italy) was eluted with 10% aqueous ethanol at the flow rate of 5 mL/min, samples elution was monitored by absorbance at 210 nm, positive fractions were pooled concentrated and lyophilized; (II) about 90–120 mg of sample were injected in a volume of 2.5 mL onto HPLC system made up by two glass columns in series (3.0 × 48 cm + 2.0 × 83 cm) filled with the resin Superdex 30 preparative grade (GE Healthcare Life Science, Milan, Italy) and a pump, a manager unit and a UV detector (KNAUER, Smartline, models 1050, 5050; and 2500, respectively) set up at 210 nm, the elution was performed with 0.25 M ammonium chloride solution at 5 mL/min, the system is managed by Clarity Chrom Preparative software, version 2.6 (Knauer, Berlin, Germany).

### 4.7. Depolymerization of CS/DS for the Isolation of HS Component

About 100 mg of danaparoid API sample was dissolved in phosphate sodium acetate buffer 50 mM pH 8, the enzyme Chondroitin ABC lyase was added to have a ratio enzyme/substrate equal to 7 mU/mg. The reaction was driven at 37 °C under magnetic stirring for 72 h.

At the end, to eliminate the enzyme by denaturation, the solution was heated at 95–100 °C for 5–10 min, after cooling the solution was filtered on 0.22 µm (Merck Millipore, Billerica, MA, USA), concentrated by reduced pressure and then purified by size-exclusion chromatography systems, applying TSK HW40S column, Superdex 30 and finally TSK HW40S column (Section 4.6).

### 4.8. Depolymerization of HS for the Isolation of CS/DS Component

About 500 mg of danaparoid API sample was dissolved in buffer 100 mM sodium acetate +10 mM calcium acetate pH 7 and incubated with the enzyme Heparinase III with a ratio of enzyme/substrate equal to 20 mU/mg, at 37 °C for 48 h under magnetic stirring. Then the solution was heated at 90–100 °C for 5–10 min for enzyme denaturation, subsequently it was cooled and filtered on 0.22 µm (Merck Millipore, Billerica, MA, USA). The solution was submitted to chemical reaction with nitrous acid twice following the procedure here described: the sample (500 mg) was dissolved in 20 mL of water, cooled to 4 °C and added to 140 mg of NaNO_2_ dissolved in 1 mL of water. The pH was adjusted to 1.7 with HCl 4% (*w*/*v*) and the solution was stirred for 20 min. Then portions of NaNO_2_ were added (140, 100 and 100 mg, respectively), after each addition the solution was stirred for 20 min. Subsequently, the pH was adjusted to 7 with NaOH 1 M and the solution was conditioned to room temperature. NaBH_4_ was added as solid (400 mg) in several portions with stirring. After 2 h, the pH solution was adjusted to 4 with HCl 4% and then neutralized with NaOH 1M. The obtained product was purified by SEC systems such as TSK HW40S column, Superdex 30 and finally TSK HW40S column (Section 4.6).

### 4.9. Depolymerization of HS for the Isolation of CS/DS Component Exhaustive Enzymatic Digestion with Lyases I, II and III

Each isolated HS fraction was depolymerized using heparinases I, II, and III according to the USP method [33]. Each sample was solubilized in water to obtain a concentration of 20 mg/mL and 20 μL (400 μg) that were then digested in sodium/calcium acetate buffer pH 7.0 by using a mixture 1:1:1 of Heparinases I, II, and III prepared by mixing a solution 0.4 IU per mL of each one. The reaction mixture was stirred (Thermo shaker TS-100, Biosan, Riga, Latvia) at 25 °C for 48 h, then each digestion solution was boiled for two minutes at 100 °C and passed through a filter having a porosity of 0.20 µm and analyzed by LC-MS.

## 5. Conclusions

For the present work an orthogonal analytical approach based on complementary techniques, allowed a compositional comparison and first structural assignment of the components of different batches of danaparoid.

The in depth studies conducted on SEC fractions and on isolated CS/DS and HS components produced much structural information that completed the characterization of the starting API samples and allowed the similarity of analyzed batches to be established, ascribing slight differences to the intrinsic heterogeneity of extractive products such as danaparoid.

From the compositional point of view, both NMR and MS data confirmed the presence of different GAGs variably distributed over fractions/molecular weights, the CS/DS component represents the higher Mw species of danaparoid. LMW heparan sulfate was confirmed to be the major component of the API samples.

NMR and LC-MS analysis underlined the fact that a majority of the analyzed species showed oxidized hexosamines as T1 variant at the RE of both CS/DS and HS chains and a remnant (Ra) of an oxidized uronic acid at the reducing end of CS/DS species. Minor further oxidation products (T2, T3, T4, and T5) were present on the HS chains. Some NMR signals were correctly assigned to GAG species by studying the isolated components.

The exemplification of species through size fractionation and isolation of components allowed a good correspondence in terms of composition with the parent danaparoid samples to be observed, supporting the robustness of the separations methods and analytical tools.

## Figures and Tables

**Table 1:** Percentage of glucosamine residues of heparan sulfate (HS) of one danaparoid sample (CAT272). A* corresponds to 3-O,N-sulfated glucosamine according with the abbreviations shown in Table S1.

A *	ANAc αred	ANAc-(I)	ANAc-(G)	ANAc-ox	ANS αred	ANS βred	ANS-(G)	ANS-(I)	ANS-(I2S)	A6S
2.2	0.9	0.3	23.4	4.4	1.7	0.4	11.4	19.3	33.5	48.4

“A *” means 3-O,N-sulfated glucosamine according with the abbreviations in Table S1.

**Table 2:** Percentage of uronic acid residues and sulfation degree of HS of one danaparoid (CAT272). (Abbreviations are in Table S1.)

I2S	I-(A6S)	I-(A6OH)	I-(ANAcox)	G-(ANS)	G-(ANAc)	Sulfation Degree
43.5	11.2	6.7	3.2	13.3	22.1	1.63

**Table 3:** Sulfate distribution (percentage) of dermatan sulfate (DS) and chondroitin sulfate (CS) of one danaparoid sample (CAT272). (Abbreviations in Table S1.)

DS		CS	
I2S	I	G-(GalNAc,4S)	G-(GalNAc,6S)
8.7	91.3	80.0	20.0

**Table 4:** Ranges of weight average molecular weight (Mw) and polydispersity (Pd) of SEC fractions of seven danaparoid Active Pharmaceutical Ingredient (API) samples.

Fractions	Mw (Da)	Pd
A	8200–8800	1.14–1.17
B	5300–5700	1.06–1.11
C	4400–4700	1.05–1.11

**Table 5:** Weight average Mw and polydispersity (Pd) of SEC fractions of danaparoid Active Pharmaceutical Ingredient (API) sample CAT277.

Fractions	A	B	C	D	E	F	G	H	I	L	M	N
Mw (kDa)	8.2	5.4	4.4	3.9	3.3	3.0	2.8	2.5	2.3	2.1	2.1	1.9
Pd	1.17	1.06	1.06	1.04	1.05	1.06	1.05	1.05	1.05	1.04	1.04	1.01

**Table 6:** Variation of dermatan sulfate and chondroitin sulfate among SEC fractions of seven API batches by qualitative observation of peculiar HSQC anomeric signals.

		A	B	C	D	E	F	G	H	I	L	M	N
CAT271	DS	+++	+++	+++	+++	++	++	++	++	+	−	−	−
	CS	+++	+++	+++	+++	++	++	++	+	−	−	−	−
CAT272	DS	+++	+++	+++	+++	++	++	++	++	++	−	−	−
	CS	+++	+++	+++	+++	++	++	++	+	+	−	−	−
CAT273	DS	+++	+++	++	++	++	++	++	+	−	−	−	−
	CS	+++	+++	++	++	++	++	−	−	−	−	−	−
CAT274	DS	+++	+++	++	++	++	++	+	−	−	−	−	−
	CS	+++	+++	++	++	+	−	−	−	−	−	−	−
CAT275	DS	+++	+++	++	++	++	++	+	−	−	−	−	−
	CS	+++	+++	++	++	+	−	−	−	−	−	−	−
CAT276	DS	+++	+++	++	++	++	+	+	−	−	−	−	−
	CS	+++	+++	++	+	−	−	−	−	−	−	−	−
CAT277	DS	+++	+++	+++	+++	++	++	++	++	+	−	−	−
	CS	+++	+++	+++	+++	++	++	++	+	−	−	−	−

Where ‘−‘ = absent; ‘+’ = traces; ‘++’ = present; ‘+++’ = more present.

**Table 7:** Main species detected in fractions A–N of 7 API samples.

Fraction	Main Species in the Woul Fraction	GAG	Species Detect at the Top of the Fraction (Pilot Study)
A	A19,10,10(T1) to A27,14,14(T1); A20,10,10(Ra) to A26,13,13(Ra)	CS/DS	-
B	A17,9,9(T1) to A21,11,11(T1); A16,8,8(Ra) to A22,11,11(Ra)	CS/DS	-
C	A13,7,7(T1) to A15,11,8(T1); A14,7,7(Ra) to A18,9,9(Ra)	CS/DS	-
D	U10,9,3 to U10,10,3; A7,10,0(T1); A11,10,6(T1) to A11,11,6(T1); U10,10,3(T1) to U10,13,3(T1)	HS	-
E	U8,8,3(T1) to U8,10,3(T1); A11,8,2(T1) to A11,14,2(T1)	HS	U8,10,2(T1); A11,14,1(T1)
F	U10,6,2(T1) to U10,8,2(T1); U10,8,1(T1) to U10,13,1(T1)	HS	U7,8,1(T5) to U7,10,1(T5); U10,8,0(T5)
G	A9,4,2(T1) ^#^ to A9,8,2(T1); A9,8,1(T1) to A9,13,1(T1)	HS	-
H	U8,6,1(T1) to U8,10,1(T1); U6,6,3(T4) to U6,8,3(T4)	HS	A9,6,2; A9,9,1 and A9,10,1; U8,6,0(T5)
I	A7,6,1 and A7,7,1; A7,5,1(T1) to A7,10,1(T1); U6,4,1 to U6,5,1; U6,6,1(T1)	HS	A7,5,2(T1); U7,4,0(T5)
L	A7,6,1; A5,5,1; A7,5,2 ^#^ to A7,6,2; U6,5,1(T1) to U6,7,1(T1); U6,6,1(T5) ^#^	HS	U6,3,0(T5) and U6,4,0(T5); A7,7,1; A7,8,0 to A7,9,0; A7,9,1
M	A5,4,1; U6,5,1 to U6,6,1; U6,6,0 to U6,9,0; A5,4,1(T1) to A5,7,1(T1); A5,7,0(T1)	HS	A5,8,1(T1)
N	A5,5,0 to A5,9,0; A5,5,1 and A5,6,1; A5,5,1(T1); U4,5,0(T3)	HS	U4,5,0(T1); U4,5,0(T2)

^#^ species not detected in one sample.

**Table 8:** Ratio percentage between disaccharides N-acetylated and N-sulfated.

Sample	Ratio % N-acetylated/N-sulfated Disaccharides
HS-CAT271	41.4
HS-CAT272	39.0
HS-CAT275	39.2
nadroparin	16.8
heparin	13.2

## Supplementary Materials

Supplementary Materials are available online.
