# Supplementary material for: Characterization of Danaparoid Complex Extractive Drug by an Orthogonal Analytical Approach
Source: Molecules. 2017 Jul 5;22(7):1116. doi: 10.3390/molecules22071116 (PMC6152146; doi:10.3390/molecules22071116)
Supplement: Supplementary file 1 [file molecules-22-01116-s001.pdf]

# Supplementary material

## Characterization of Danaparoid complex extractive drug by an orthogonal analytical approach

**Cristina Gardini [1], Elena Urso [2], Marco Guerrini [2], René van Herpen [3], Pauline de Wit [3] and Annamaria Naggi [2,*]**

[1] Centro Alta Tecnologia Istituto di Ricerche Chimiche e Biochimiche G. Ronzoni S.r.l., via G. Colombo 81, Milan, 20133, Italy; gardini@cat-ronzoni.it (C.G.)
[2] Istituto di Ricerche Chimiche e Biochimiche G. Ronzoni S.r.l., via G. Colombo 81, Milan, 20133, Italy; urso@ronzoni.it (E.U.); guerrini@ronzoni.it (M.G.); naggi@ronzoni.it (A.N.)
[3] Aspen Oss B.V., Kloosterstraat 6, 5349 AB Oss, Netherlands; rvanherpen@nl.aspenpharma.com (R.v.H.); pdewit@nl.aspenpharma.com (P.d.W.)
* Correspondence: naggi@ronzoni.it; Tel.: +39 02 70641626

Contents:

Table S1: Abbreviations of assigned fragments.

Table S2: Results summary table regarding CS/DS fractions: assignment of main signals.

Table S3: Comparison of the main mass signals between Danaparoid CAT272 and its HS fraction.

Figure S1: $^1$H NMR spectrum of Danaparoid samples: expansion of the acetyl region.

Figure S2: LC-MS chromatograms of a Danaparoid sample CAT277, the LC method was composed by an initial isocratic step followed by linear gradient: (a) Total Ion Chromatogram (TIC); (b) Base Peak Chromatogram (BPC).

Figure S3: LC-MS chromatograms: (a) repeatability of CAT277; (b) interday precision of CAT277; (c) interday precision of CAT271.

Figure S4: Acetyl region of proton spectra: comparison between (a) one Danaparoid sample CAT277; (b) its ChABC digestion product CAT469.

Figure S5: MS/MS spectrum of mass signal at m/z 466.0 (z -2) attributed to ΔU4,2,2(T1) at the Collision Energy (CE) of 25 eV and the assignment of fragments.

Figure S6: MS/MS spectrum of mass signal at m/z 524.0 (z -2) attributed to ΔU5,2,2(Ra) at the Collision Energy (CE) of 25 eV and the assignment of fragments.

Figure S7(a): Anomeric region of SEC fractions A-F of one Danaparoid sample (CAT272).

Figure S7(b): Anomeric region of SEC fractions G-N of one Danaparoid sample (CAT272).

**Table S1.** Abbreviations of assigned fragments.

| Residue | Abbreviation |
|---|---|
| H2/C2 N-sulfated glucosamine | ANS |
| H2/C2 3-O,N-sulfated glucosamine | A2* |
| H2/C2 N-acetyl glucosamine | ANAc |
| H6/C6 6-O-unsulfated glucosamine | A6OH |
| H6/C6 6-O-sulfated glucosamine | A6S |
| H1/C1 N-sulfated glucosamine *linked to* β-D-glucuronic acid | ANS-(G) |
| H1/C1 3-O,N-sulfated glucosamine | A1* |
| H1/C1 N-sulfated glucosamine *linked to* 2-O-sulfo α-L-iduronic acid+ H1/C1 N-acetyl glucosamine *linked to* β-D-glucuronic acid | Atot |
| H1/C1 N-sulfated glucosamine *linked to* α-L-iduronic acid | ANS-(I) |
| H1/C1 N-acetyl glucosamine *linked to* α-L-iduronic acid | ANAc-(Ix) |
| H1/C1 reducing N-sulfated α-D-glucosamine + H1/C1α-D-glucosamine | ANSαred+$ANH_2$ |
| H1/C1 reducing N-acetyl α-D- glucosamine | ANAcαred |
| H1/C1 reducing N-sulfated β-D-glucosamine | Aβred |
| H1/C1 2-O-sulfo α-L-iduronic acid | I2S |
| H1/C1 α-L-iduronic acid *linked to* H6/C6 N sulfate /acetylated 6-O-sulfated α-D-glucosamine | I-(A6S) |
| H1/C1 α-L-iduronic acid *linked to* H6/C6 of N sulfate /acetylated α-D-glucosamine | I-(A6OH) |
| H1/C1 β-D-glucuronic acid *linked to* N-sulfated α-D-glucosamine | G-(ANS) |
| H1/C1 β-D-glucuronic acid *linked to* N-acetyl α-D-glucosamine | G-(ANAc) |
| Oxidized N-acetylglucosamine | ANAc-ox |
| H1/C1 β-D-glucuronic acid *linked to* 4-O-sulfo-N-acetylgalactosamine | G-(GalNAc,4S) |
| H1/C1 β-D-glucuronic acid *linked to* 6-O-sulfo-N-acetylgalactosamine | G-(GalNAc,6S) |
| H1/C1 α-L-iduronic acid *linked to* β-N-acetylgalactosamine | I_DS |
| H1/C1 2-O-sulfo α-L-iduronic acid *linked to* β-N-acetylgalactosamine | I2S_DS |
| H2/C2 β-N-acetylgalactosamine (DS+CS) | GalNAc |
| H1/C1 α-L-iduronic acid *linked to* Oxidized N-acetylglucosamine | I-(ANAcox) |
| Oxidized N-acetyl galactosamine | GalNAc-ox |
| Oxidized HS ox1 | ox1 |
| Oxidizex HS ox2 | ox2 |
| Non reducing N- acetyl galactosamine | GalNAc_NR |

**Table S2.** Results summary table regarding CS/DS fractions: assignment of main signals.

| Peak N. | Experimental m/z (z) | MW | Assigned composition | Peak N. | Experimental m/z (z) | MW | Assigned composition |
|---|---|---|---|---|---|---|---|
| 1 | 903.7(-2) | 1809.4 | - | 14 | 1429.7(-3) | 4292.1 | A16,8,8(Ra) |
| | 634.6(-2) | 1271.2 | A6,2,3(Ra) | 15 | 1587.1(-3) | 4764.3 | A17,9,9(T1) |
| 2 | 991.7(-2) | 1985.4 | - | 16 | 1625.8(-3) | 4880.4 | A18,9,9(Ra) |
| 3 | 910.7(-2) | 1823.4 | A7,4,4(T1) | 17 | 1783.2(-3) | 5352.6 | A19,10,10(T1) |
| 4 | 968.7(-2) | 1939.4 | A8,4,4(Ra) | 18 | 1821.9(-3) | 5468.7 | A20,10,10(Ra) |
| 5 | 1056.7(-2) | 2115.4 | - | 19 | 1451.9(-4) | 5811.6 | A21,11,11(T1) |
| | 1285.8(-2) | 2573.6 | - | 20 | 1448.7(-4) | 5798.8 | A22,11,11(Ra) |
| 6 | 1204.8(-2) | 2411.6 | A9,5,5(T1) | 21 | 1598.9(-4) | 6399.6 | A23,12,12(T1) |
| 7 | 1262.8(-2) | 2527.6 | A10,5,5(Ra) | 22 | 1628.2(-4) | 6516.8 | A24,12,12(Ra) |
| 8 | 1415.4(-2) | 2832.8 | - | 23 | 1746.5(-4) | 6990 | A25,13,13(T1) |
| 9 | 1563.5(-2) | 3129.0 | A11,6,6(T1) | 24 | 1775.3(-4) | 7105.2 | A26,13,13(Ra) |
| 10 | 951.5(-3) | 2857.5 | - | 25 | 1893.6(-4) | 7578.4 | A27,14,14(T1) |
| 11 | 1195.0(-3) | 3588.0 | A13,7,7(T1) | 26 | 1922.6(-4) | 7694.4 | A28,14,14(Ra) |
| 12 | 1233.7(-3) | 3704.1 | A14,7,7(Ra) | 27 | 2069.6(-4) | 8282.4 | A30,15,15(Ra) |
| 13 | 1304.9(-3) | 3917.7 | - | | | | |

**Table S3.** Comparison of the main mass signals between Danaparoid CAT272 and its HS fraction.

| HS-Danaparoid | | | CAT272 | | |
|---|---|---|---|---|---|
| Peak N. | Experimental m/z | Assigned composition | Peak N. | Experimental m/z | Assigned composition |
| 1 | 568.6062 | A5,2,3* | - | | |
| 2 | 647.6176 | ΔU6,2,3* | - | | |
| 3 | 648.5645 | A5,4,3* | - | | |
| 4 | 695.5908 | ΔU6,3,3(T1)* | a | 625.5269 | A5,5,0 |
| 5 | 727.5733 | ΔU6,4,3* | - | | |
| 6 | 713.6211 | ΔU7,2,3(Ra)* | b | 505.0112 | U4,4,0 |
| 7 | 614.5498 | A5,4,1(T1) | c | 614.5497 | A5,4,1(T1) |
| 8 | 702.5671 | U6,4,1(T1) | d | 702.5654 | U6,4,1(T1) |
| 9 | 654.529 | A5,5,1(T1) | e | 654.5277 | A5,5,1(T1) |
| 10 | 742.5434 | U6,5,1(T1) | f | 742.5419 | U6,5,1(T1) |
| 11 | 967.6087 | A7,7,1(T1) | g | 967.6096 | A7,7,1(T1) |
| 12 | 1055.6233 | U8,7,1(T1) | h | 1055.627 | U8,7,1(T1) |
| 13 | 1072.1597 | A7,8,1(T1) | i | 1072.1602 | A7,8,1(T1) |
| 14 | 1160.1763 | U8,8,1(T1) | l | 1160.1752 | U8,8,1(T1) |
| 15 | 1176.7109 | A7,9,1(T1) | m | 1176.7131 | A7,9,1(T1) |

* These components could be CS/DS fragments not completely separated from the HS fraction.

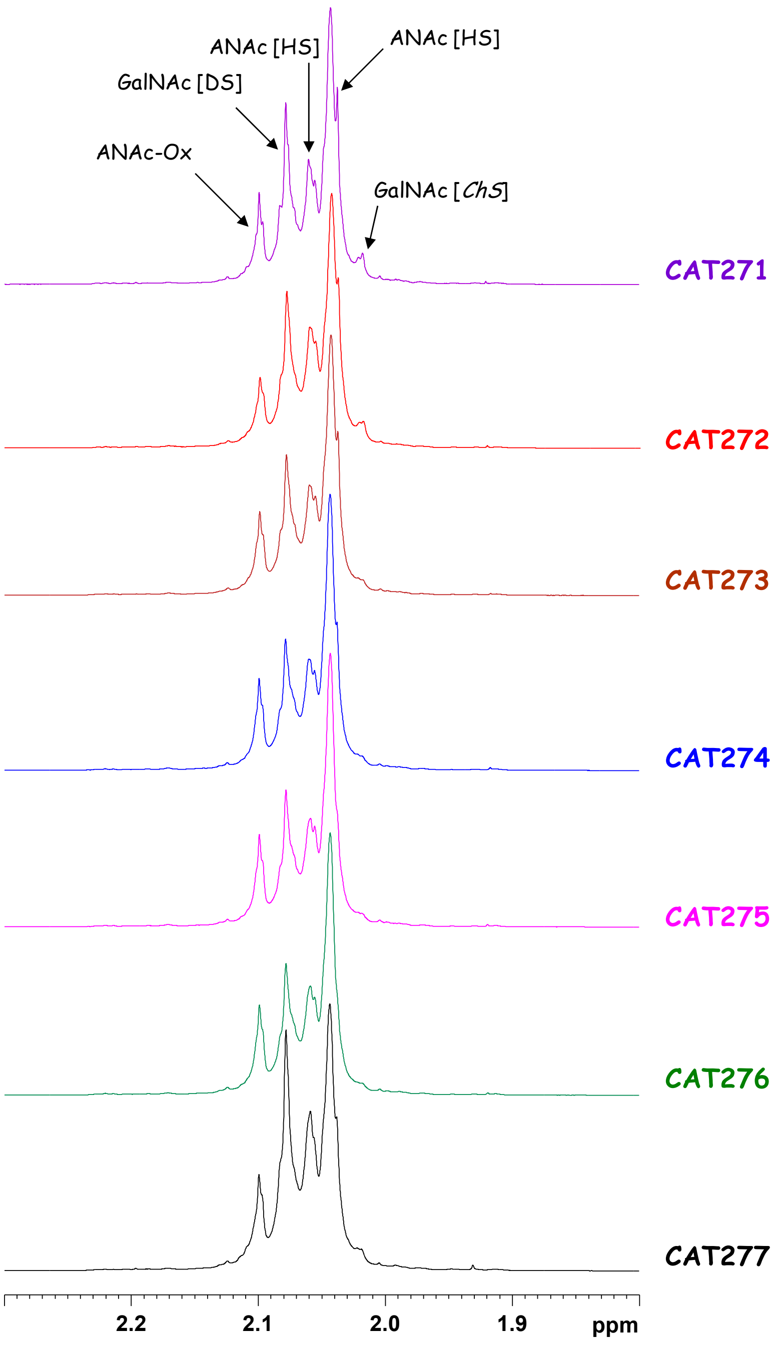


**Figure S1.** $^{1}$H NMR spectrum of a Danaparoid samples: expansion of the acetyl region.

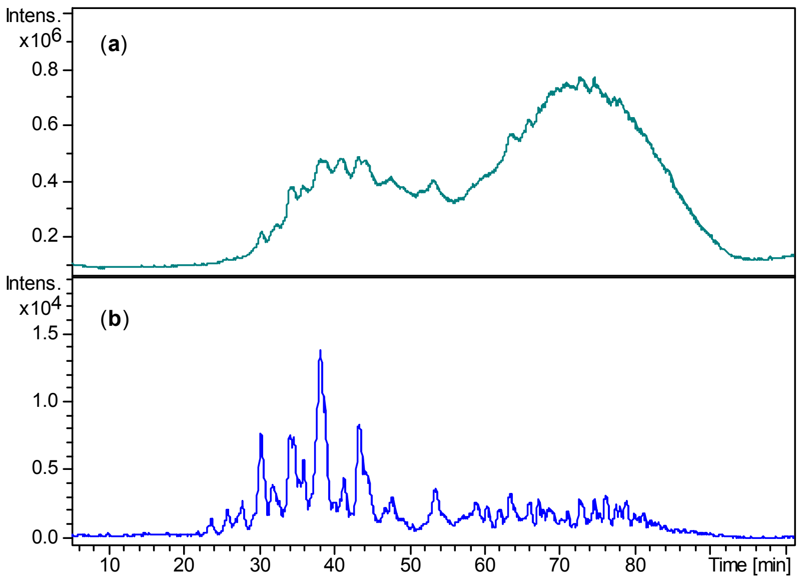


**Figure S2.** LC-MS chromatograms of a Danaparoid sample CAT277, the LC method was composed by an initial isocratic step followed by linear gradient: (a) Total Ion Chromatogram (TIC); (**b**) Base Peak Chromatogram (BPC).

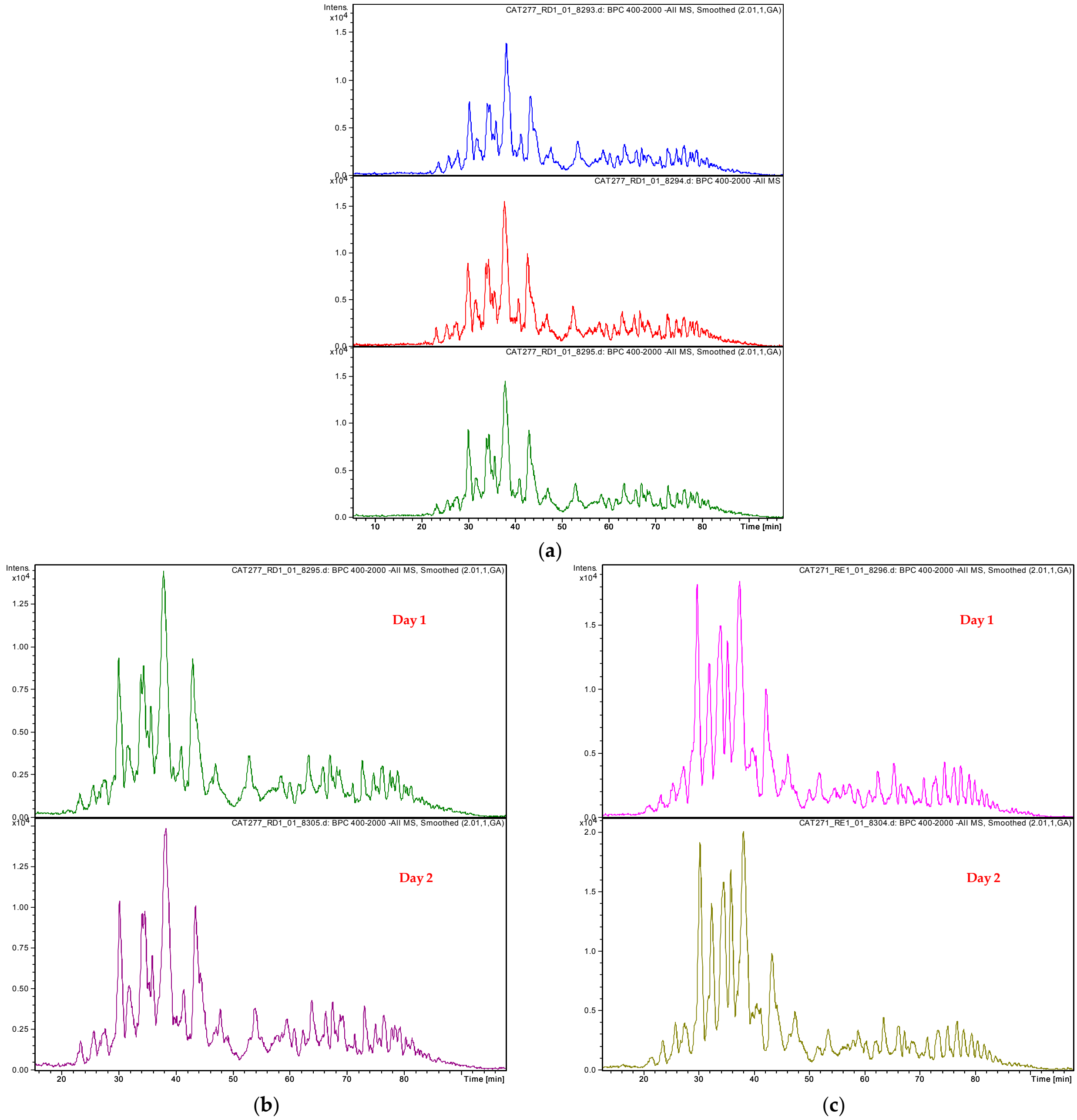


**Figure S3**. LC-MS chromatograms: (a) repeatability of CAT277; (b) interday precision of CAT277; (c) interday precision of CAT271.

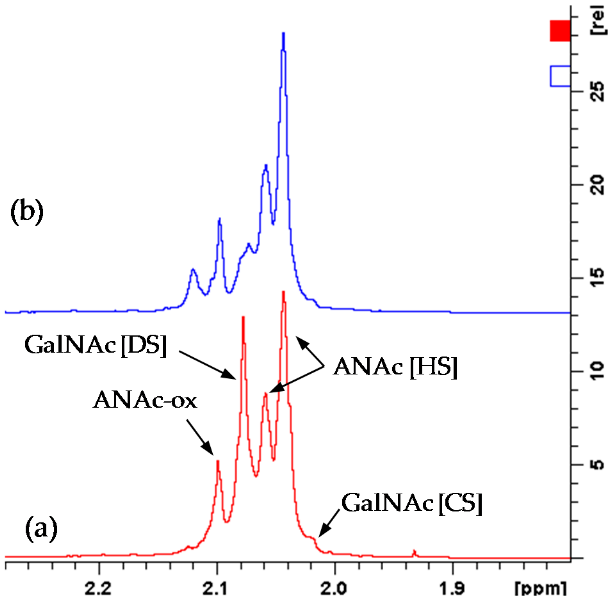


**Figure S4.** Acetyl region of proton spectra: comparison between (**a**) one Danaparoid sample CAT277; (**b**) its ChABC digestion product CAT469.

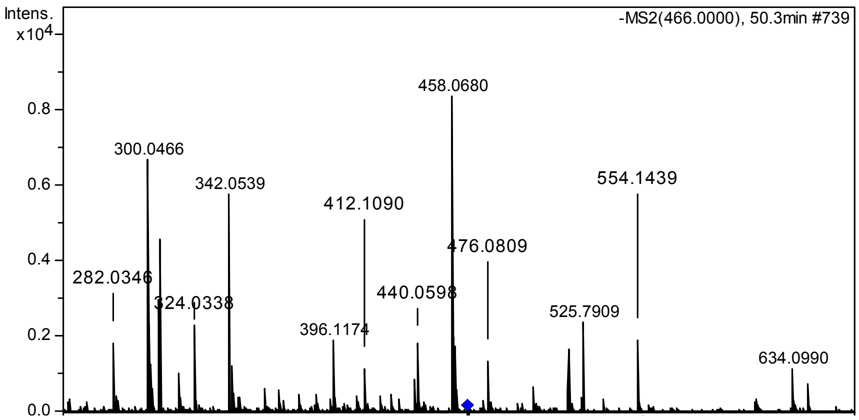


| m/z (-z) | M | Structure hypothesis |
|---|---|---|
| **466.0 (-2)** | **934** | **ΔU4,2,2+O** |
| 458.1 (-1) | 459 | ΔU2,1,1 |
| 440.0 (-1) | 441 | ΔU2,1,1( – $H_2O$) |
| 412.1 (-1) | 413 | **U-ANAc +O** |
| 396.1 (-1) | 397 | U-ANAc |
| 342.1 (-1) | 343 | 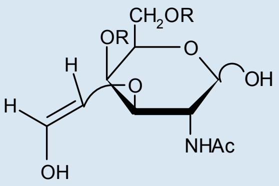  where R = $SO_3$, H |
| 316.0 (-1) | 317 | **ANAc, S +O** |
| 300.0 (-1) | 301 | ANAc, S |
| 282.0 (-1) | 283 | ANAc, S (– $H_2O$) |
| 554.1 (-1) | 555 | ΔU-ANAc-U |
| 634.1 (-1) | 635 | ΔU-ANAc-U (+$SO_3$) |

**Figure S5.** MS/MS spectrum of mass signal at m/z 466.0 (z -2) attributed to ΔU4,2,2(T1) at the Collision Energy (CE) of 25 eV and the assignment of fragments.

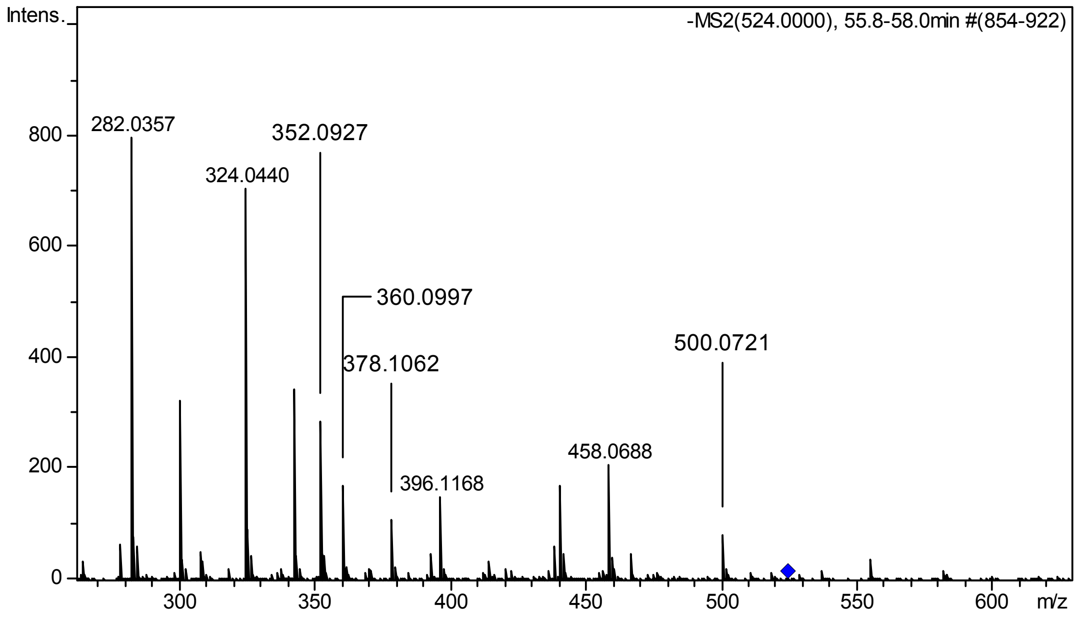


| m/z (-z) | M | Structure hypothesis |
|---|---|---|
| **524.0 (-2)** | **1050** | **ΔU4,2,2(+$C_4H_4O_5$)** |
| 500.1 (-1) | 501 | - |
| 458.1 (-1) | 459 | ΔU2,1,1 |
| 440.0 (-1) | 441 | ΔU2,1,1(-$H_2O$) |
| 396.1 (-1) | 397 | U-ANAc |
| 378.1 (-1) | 379 | ΔU-ANAc |
| 360.1 (-1) | 361 | ΔU-ANAc (-$H_2O$) |
| 352.1 (-1) | 353 | ANAc (+$C_4H_4O_5$) |
| 342.1 (-1) | 343 | 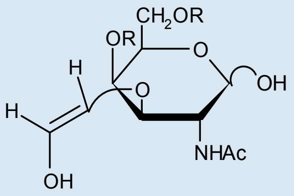  where R = $SO_3$, H |
| 324.1 (-1) | 325 | - |
| 536.1 (-1) | 537 | ΔU-ANAc-U(-$H_2O$) |
| 554.1 (-1) | 555 | ΔU-ANAc-U |

**Figure S6.** MS/MS spectrum of mass signal at m/z 524.0 (z -2) attributed to ΔU5,2,2(Ra) at the Collision Energy (CE) of 25 eV and the assignment of fragments.

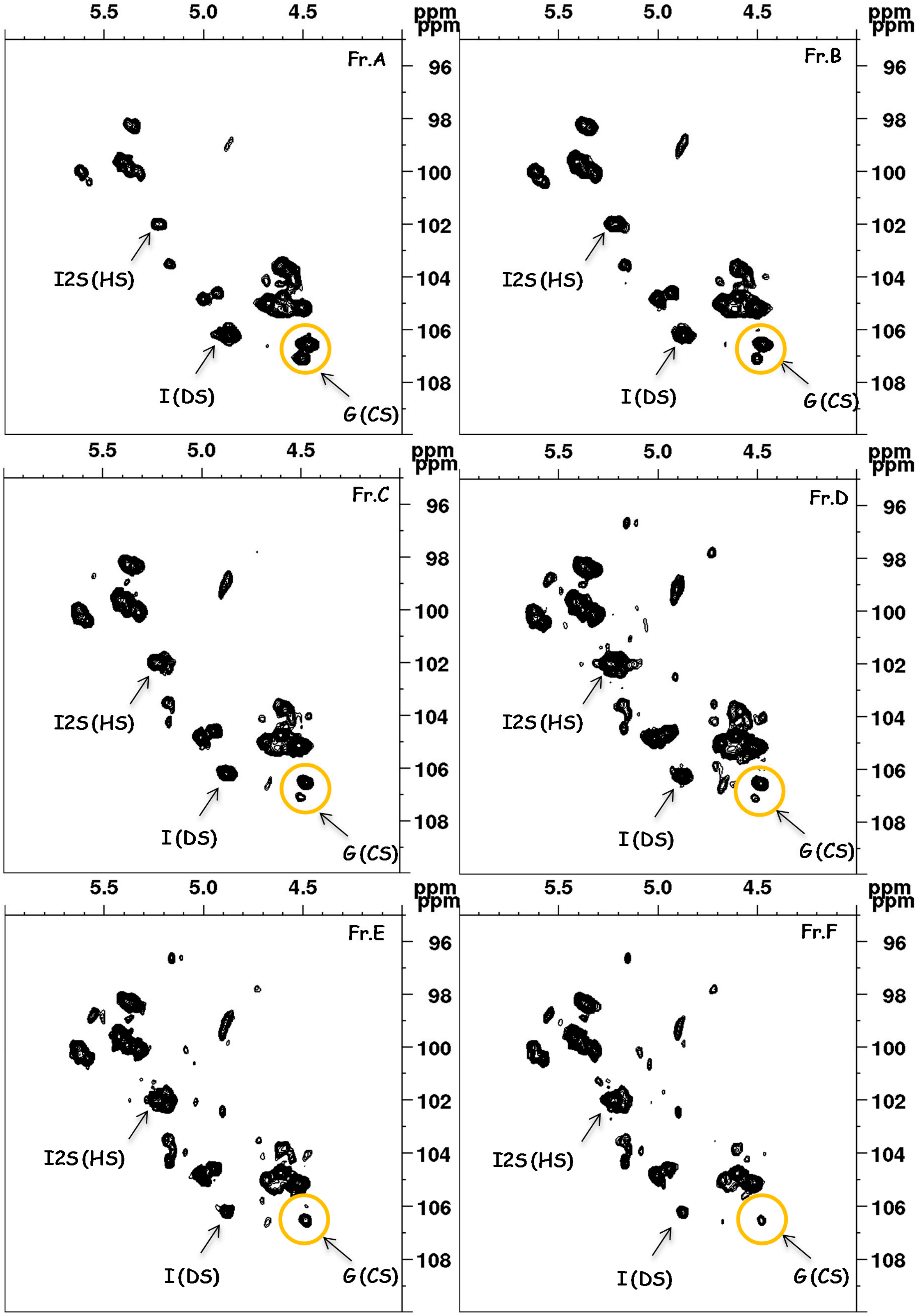

**Figure S7(a).** Anomeric region of SEC fractions A-F of one Danaparoid sample (CAT272).

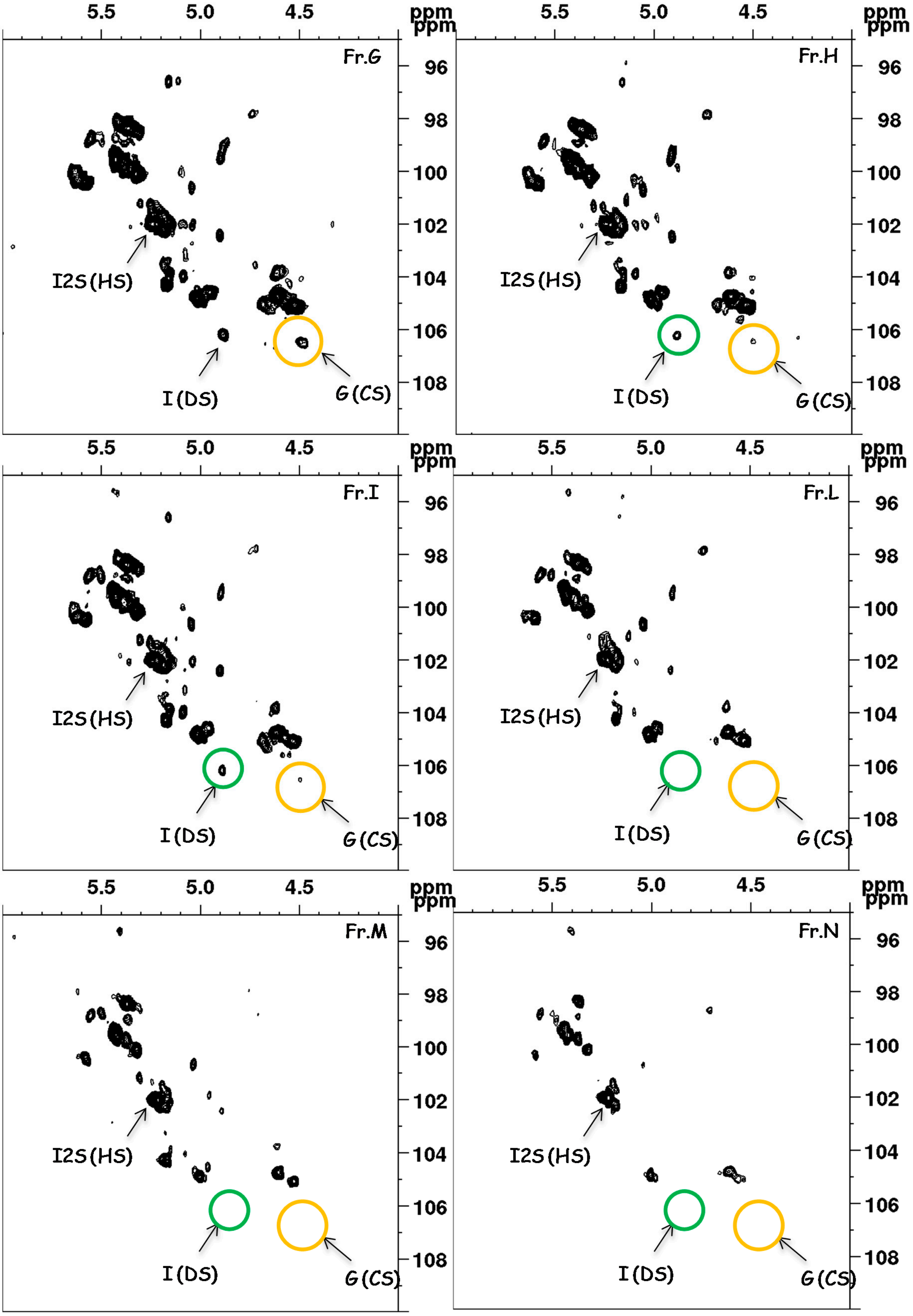


**Figure S7(b).** Anomeric region of SEC fractions G-N of one Danaparoid sample (CAT272).
